# Recent progress in the structure–activity relationship of antibacterial peptoids

**DOI:** 10.1039/d6ra07372g

**Published:** 2026-09-21

**Authors:** Ghayah Bahatheg, Tope Abraham Ibisanmi, David StC. Black, Mark D. P. Willcox, Naresh Kumar

**Affiliations:** a Department of Chemistry, College of Science, University of Jeddah P. O. Box 80327 Jeddah 21589 Saudi Arabia; b School of Chemistry, The University of New South Wales (UNSW) Sydney NSW 2052 Australia t.ibisanmi@unsw.edu.au n.kumar@unsw.edu.au; c School of Optometry and Vision Science, The University of New South Wales (UNSW) Sydney NSW 2052 Australia

## Abstract

Antimicrobial peptides (AMPs) have garnered interest as a promising therapeutic option due to their high potency against multidrug-resistant bacterial strains, primarily attributed to their amphiphilic cationic properties, which enable them to directly interact with bacterial cells *via* the negatively charged cell membrane, ultimately leading to bacterial cell death. The clinical use of AMPs is limited by cytotoxicity arising from poor selectivity between microbial and host membranes. Their susceptibility to enzymatic degradation and high manufacturing costs further constrain their use. These drawbacks of AMPs created an urgent need to find an alternative class of antibacterial molecules with enhanced structural features known as peptidomimetics. Peptoids are a new class of peptidomimetics that mimic the structure of AMPs, overcoming several of these drawbacks through resistance to proteolysis and efficient, low-cost synthesis of short sequences. This review provides a comprehensive overview of recent progress in the structure activity relationship of antibacterial peptoids, and critically evaluates how structural features influence biological outcomes.

## Abstract 2

Drawbacks of antimicrobial peptides (AMPs) have prompted the development of peptidomimetics that incorporate structural modifications while retaining key AMP mechanisms. Peptoids are promising peptidomimetics that mimic key AMP features while offering greater resistance to enzymatic degradation.

## Graphical abstract

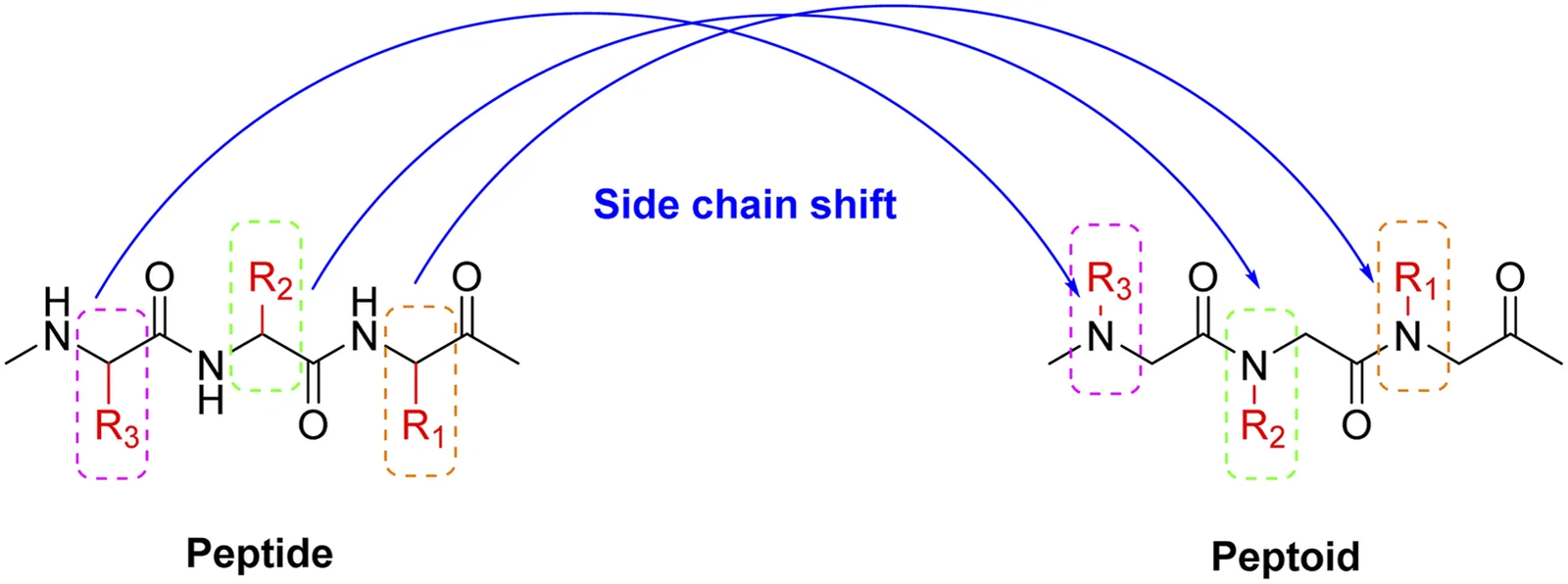

## 1 Introduction

Traditional antibiotics were the first reported drugs with potential activity against pathogenic bacteria. Despite their effectiveness, they have failed to fulfill their mission as trusted antibacterial drugs due to bacterial multidrug resistance.^1,2^ Natural AMPs are derived from living organisms and have emerged as important leads and mechanistic inspirations for next-generation anti-infective design,^1^ particularly in the context of multidrug resistance.^3–5^ These AMPs have unique biological features that enable them to fight and kill bacterial cells, and which has kept them as a good alternative to antibiotics for years.^6,7^ Most AMPs share similar structural and activity features, including the presence of cationic and hydrophobic units with/without backbones in various conformations, such as short, long, linear, and cyclic structures.^6^ These amphiphilic cationic AMPs utilize their structural features to directly kill bacterial cells through interaction between their positive net charge (cationic group) and the negatively charged cell membrane.^8–10^ Despite these characteristics of AMPs, their use is limited due to their toxicity, instability in enzymatic environments, high production costs and low yields.^11^

The AMPs' drawbacks prompted the synthesis of peptidomimetics, which can have various modifications while retaining their unique mode of action. Peptoids are a promising antibacterial drug of peptidomimetics^12^ that mimic the structure of AMPs with greater stability against enzymatic degradation and a lower production cost.^13,14^

Peptoids, or poly-*N*-substituted glycines, are a new class of synthetic antimicrobial molecules, meaning they do not occur naturally.^15^ Peptoids were introduced at Chiron Corporation in Emeryville, California, in the early 1990s as a modular approach to drug discovery, with the enabling submonomer solid-phase synthesis reported in 1992.^16^ Over subsequent decades, peptoids showed their potential as essential molecules in drug discovery and many pharmaceutical and biotechnology applications.^17,18^ Peptoids mimic the structure and role of peptides but avoid their drawbacks. Peptides exemplify a unique therapeutic design, situated structurally between small compounds and proteins, yet differing pharmaceutically and chemically from both.^19^

A great amount of knowledge has been accumulated over the last ten years about the interplay between the structure of peptoids and their antibacterial efficacy, selectivity, and toxicity.^1^ These tremendous developments comprise, among others, a thorough understanding of the impact of cationic charge, hydrophobicity, amphiphilicity, side chain chemistry, and conformational control in different peptoid architectures on the antibacterial activity.

Despite this rapid growth, the structure activity relationship landscape of antibacterial peptoids remains fragmented across studies that focus on specific scaffolds, modifications, or biological targets. A consolidated and critical review of recent findings is therefore needed to identify unifying design principles and to highlight emerging trends that guide rational peptoid optimization. This literature review aims to integrate recent progress in the structure activity relationship of antibacterial peptoids, critically evaluate how structural features influence biological outcomes, and outline current challenges and future directions for translating peptoid based antimicrobials toward clinical relevance.

## 2 Fundamentals of peptoids

Peptoids were developed to emulate the characteristics of AMPs and thus, their structure and function also address the main shortcomings of AMP. Similar to AMPs, most antimicrobial peptoids possess cationic and amphipathic properties. Although they do not form classical α-helical conformations due to the absence of backbone hydrogen bonding, they are well known to adopt stable helix-like structures, with hydrophobic and charged moieties segregated.^1^ The net positive charge and the balance of hydrophobic and hydrophilic features in most peptoids are derived from design principles established for AMPs, giving rise to cationic and amphipathic structures.^20^ However, the peptoid backbone is fundamentally different.

**Fig. 1:**
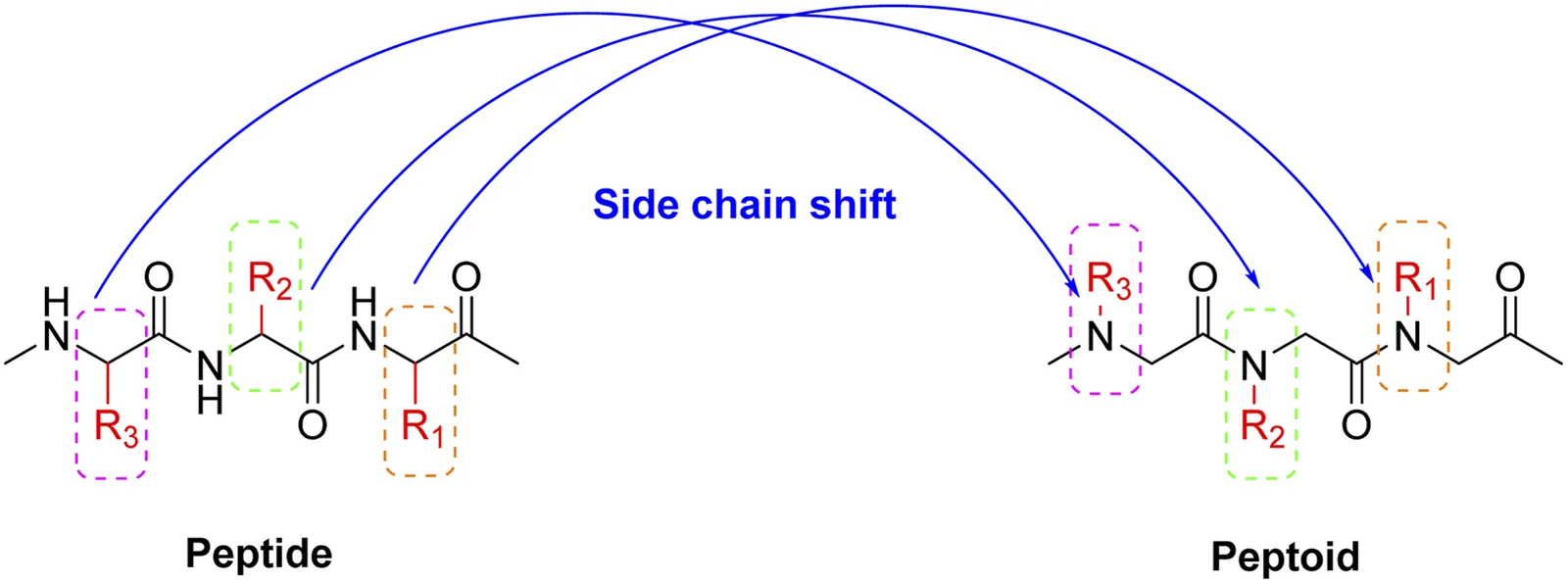
The difference between peptide and peptoid backbones.

**Fig. 2:**
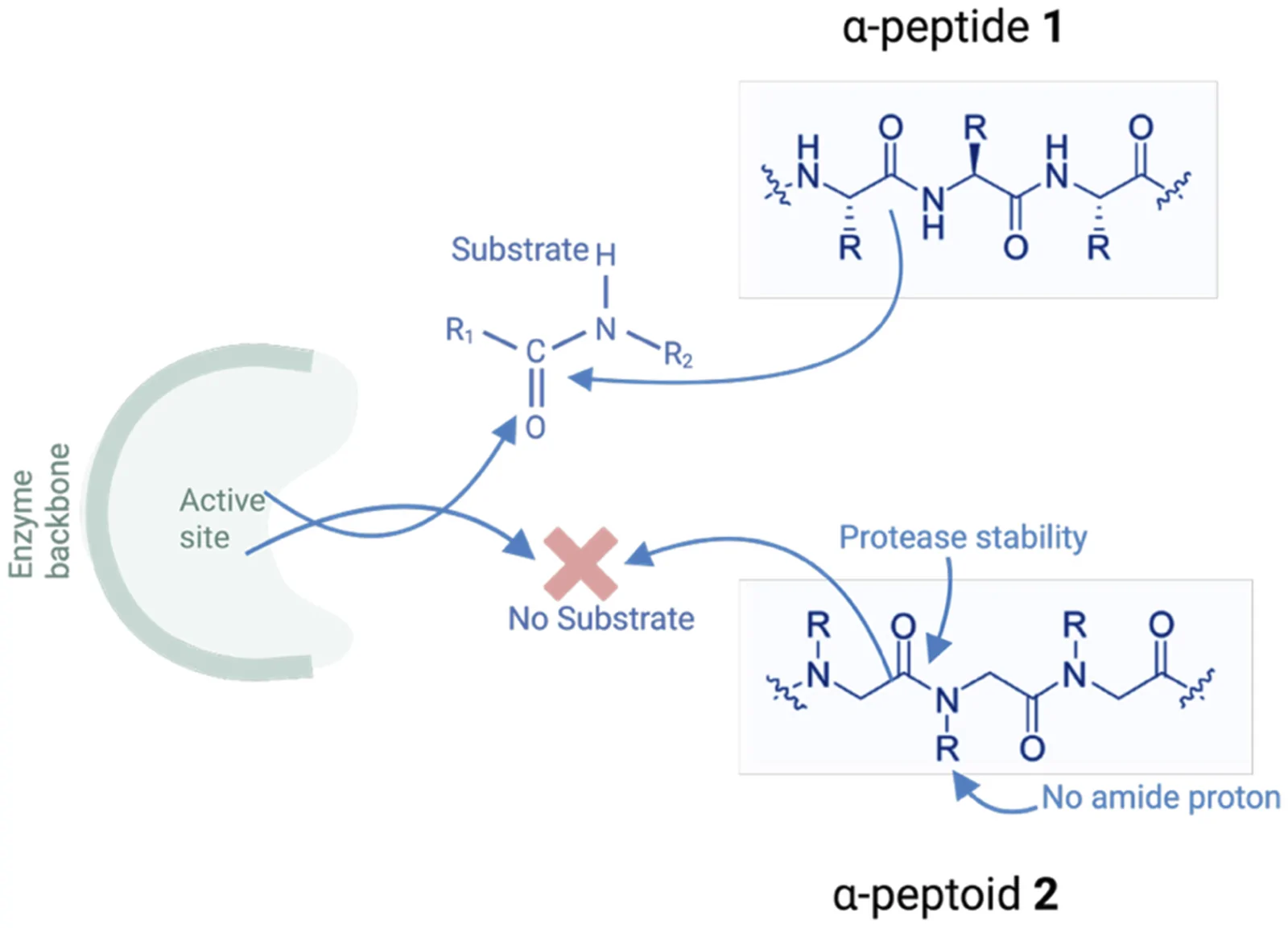
Peptoids exhibit greater protease resistance compared to peptides. Peptides contain backbone amide protons that facilitate recognition and cleavage by proteases, whereas peptoids lack these features, which is thought to contribute to their enhanced proteolytic stability, although the exact mechanism remains unclear.

The fundamental backbone of peptoids is typical of peptides, except in two respects (Fig. 1). Firstly, in each peptoid unit, the side chain is linked to the amide nitrogen atom; in contrast, the side chain in the peptide is connected to the alpha-carbon atom. Secondly, the nature of amino acid units restricts the activity and diversity of peptides, whereas in peptoids, there are no limitations on side-chain types (Fig. 1).^16,21–23^ Unlike peptides, the absence of hydrogen atoms linked to the amide nitrogen in peptoids inhibits the formation of intra- and inter-molecular hydrogen bonds, which creates a new family of molecules (Fig. 2).^24^ The loss of backbone hydrogen bonding in peptoids together with their non-natural backbone structure, enhances their resistance to proteolytic degradation despite their conformational flexibility, as demonstrated in an *in vitro* proteolytic study conducted by Zuckermann and coworkers.^13,25^

**Fig. 3:**
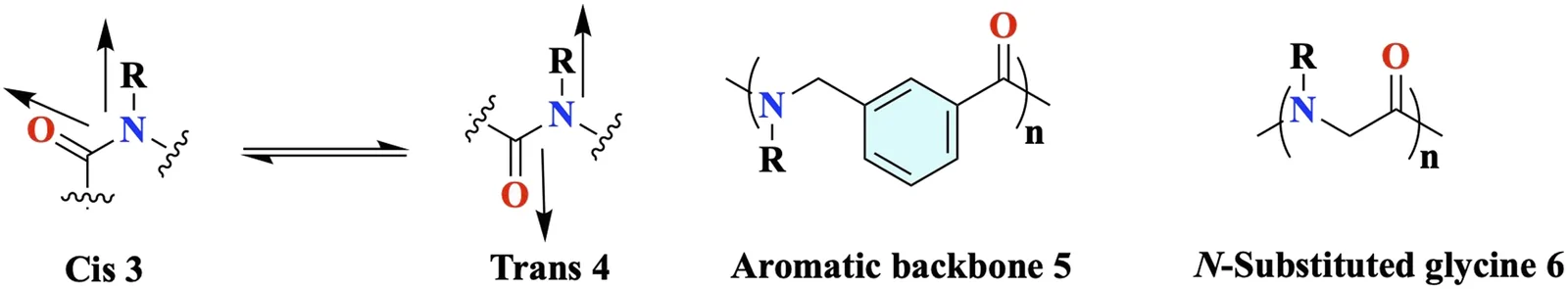
Structural features of peptoids, including *cis*/*trans* amide rotamers and representative backbone architectures. Arrows indicate interconversion between *cis* and *trans* conformations.

One of the key contributors to the chemical diversity of peptoids is the sub-monomer synthesis method developed by Zuckermann and coworkers in 1992.^15^ This method involves iterative steps of acylation of the resin-bound amine followed by nucleophilic substitution by substituted primary amines, and these steps are repeated several times to produce the desired peptoid before cleaving the resin.^26^ Early structural studies by Zuckermann and co-workers demonstrated that peptoids exhibit greater backbone flexibility than peptides due to the absence of backbone hydrogen bonding and increased rotational freedom about the tertiary amide bond.^15^ This conformational flexibility allows peptoids to populate both *cis* and *trans* amide rotamers (Fig. 3), with the equilibrium and interconversion between these states having been further investigated in subsequent studies.^10^

Apart from the conventional peptoids 6, extended peptoids 5 incorporate aromatic units directly into the backbone, increasing the distance between amide bonds and introducing rigidity.^27^ This backbone extension restricts conformational freedom and promotes more defined spatial arrangement of side chains, allowing improved control over amphipathic patterning and charge presentation compared with standard peptides.^28^ Modification of the peptoid backbone eliminates classical backbone hydrogen bonding and reduces protease recognition, while maintaining chemical diversity through side chain substitution at the nitrogen.

## 3 Physicochemical determinants of antibacterial activity

### 3.1 Cationicity and positive charge

**Fig. 4:**
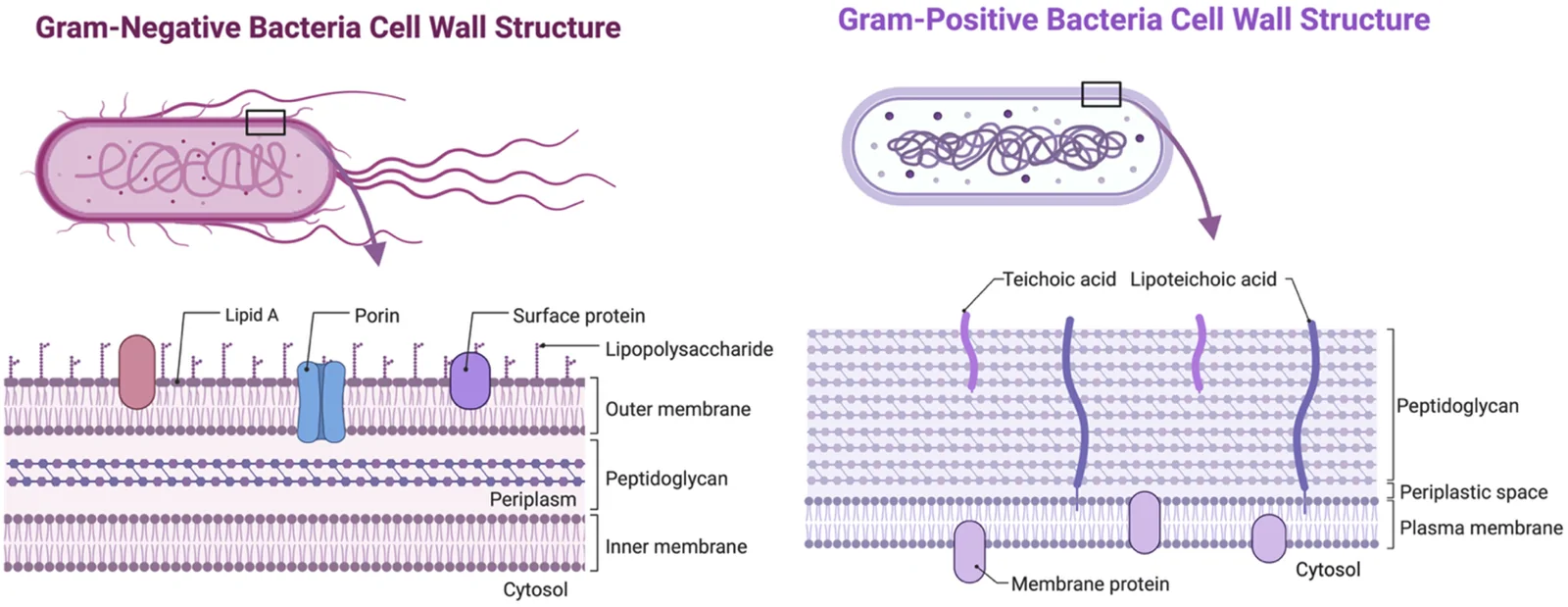
Schematic representation of bacterial cell wall structures in Gram-positive and Gram-negative bacteria. Created with BioRender.

A net positive charge is a defining hallmark of most membrane-active antibacterial peptides and peptoids. Bacterial cytoplasmic membranes and cell envelopes are enriched in anionic components including phosphatidylglycerol and cardiolipin in the inner membrane, lipopolysaccharide (LPS) in Gram-negative outer membranes, and teichoic acids in Gram-positive cell walls whereas mammalian plasma membranes are dominated by zwitterionic phosphatidylcholine, sphingomyelin and cholesterol with only limited anionic content (Fig. 4). This electrostatic asymmetry highlights the preferential binding of cationic agents to bacteria over host cells. Cationic peptoids exploit this same principle. Positively charged side chains drive initial attraction to the negatively charged bacterial surface, allowing local accumulation at the membrane interface. Once enriched at the bacterial cell surface through electrostatic attraction, hydrophobic residues within antimicrobial peptoids can partition into the lipid bilayer, triggering membrane thinning, pore formation, or more extensive membrane destabilization events. As observed for host-defense AMPs, this initial electrostatic engagement in peptoids is primarily mediated by cationic side chains.

**Fig. 5:**
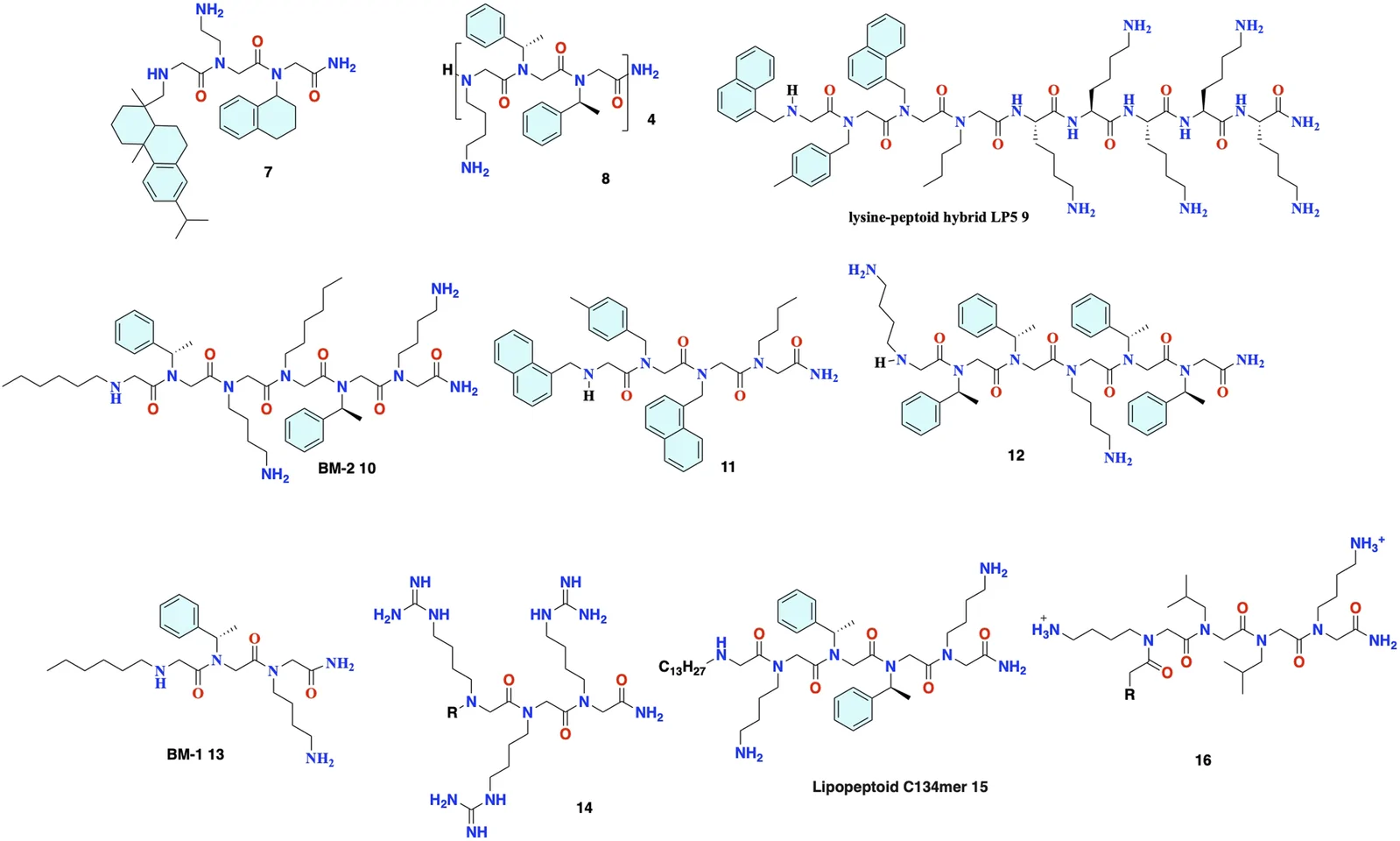
Selected examples of short antibacterial peptoids.

The first report on antibacterial short cationic peptoids was in 1998. Among a combinatorial library of approximately 840 compounds, CHIR29498 (7) showed the highest antimicrobial activity (Fig. 5).^1,29^

In most antibacterial peptoid designs, positive charge is introduced through lysine-mimetic primary aminoalkyl side chains. These residues bear primary ammonium groups that are fully protonated at physiological pH, providing stable cationic charge and the ability to engage in electrostatic and hydrogen-bonding interactions with anionic phospholipids. Their straightforward synthesis *via* the submonomer route and compatibility with solid-phase methods facilitated construction of large libraries. One of the widely known lysine base peptoid is LP5 9, LP5 may have a dual mode of action against *Staphylococcus aureus*. At sub-MIC concentrations, LP5 binds to DNA and inhibits macromolecular synthesis and growth, whereas at higher concentrations near or above the MIC, it disrupts the bacterial membrane.^30^ Another example is the short cationic peptoid BM2 10, designed by expanding the BM1 13 sequence which has demonstrated activity against *M. bovis* BCG, the laboratory strain *M. tuberculosis* H37Rv, and the multidrug-resistant clinical isolate *M. tuberculosis* CSU87.

Further, arginine-type monomers have been reported to enhance biological activity when incorporated into peptoid sequences, although this enhancement is often accompanied by increased toxicity toward mammalian cells.^1,31^ Further, Guanidinium groups can form stronger electrostatic and hydrogen-bonding interactions with zwitterionic lipids compared with primary ammonium groups, reducing the charge-based discrimination between bacterial and host membranes.^1^ Another study by Wender and co-workers demonstrated that oligomers rich in guanidinium moieties can cross cell membranes *via* mechanisms that involve multidentate hydrogen bonding with anionic headgroups and glycosaminoglycans.^32^ Derivatives of peptoids (Fig. 5) have been designed by Haldar's group using naphthyl, chloroanthracenyl, phenyl, and lysine amino acids bearing different alkyl chains on the amide NH group. The most active molecule was chloroanthracenyl peptoid with MICs of 2.9 µg mL^−1^ against *Escherichia coli* and 2.2 µg mL^−1^ against *Staphylococcus aureus*.^33^

**Fig. 6:**
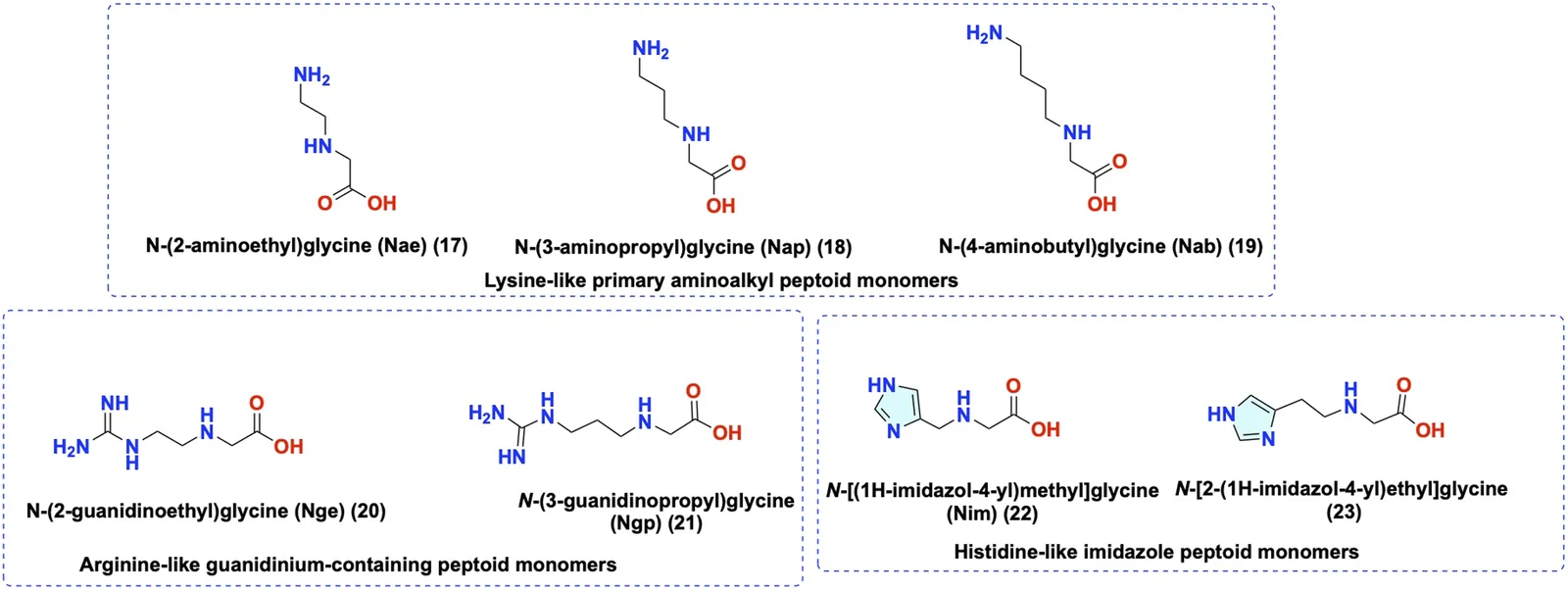
Cationic side-chain derivatives illustrating structural variations used to modulate net positive charge.

Less commonly, histidine-mimetic imidazole-containing side chains are incorporated for *N*-(imidazolylmethyl)glycine (Nim) 22, and *N*-(imidazole-ethyl)glycine 23, offering pH-dependent protonation that may confer environment-responsive activity under acidic conditions typical of infection or inflammation sites.^20,28^ However, both the chemical identity of the cationic group and the number and distribution of these groups along the sequence have marked effects on antibacterial potency and selectivity. Once concentrated at the surface, hydrophobic residues can partition into the bilayer, leading to membrane thinning, pore formation or other disruptive events (Fig. 6).

To date, most cationic peptoids reported in the literature consist exclusively of either guanidinium-functionalized (arginine-type) or amino-functionalized (lysine-type) monomers. In general, replacing lysine-type residues with arginine-type analogues tends to increase antibacterial potency, particularly against Gram-negative bacteria and mycobacteria, but often at the cost of higher toxicity to mammalian cells. In translating this concept to peptoid design, Bolt and Cobb developed a robust synthetic approach that enables the incorporation of both lysine-type and arginine-type cationic monomers within a single sequence, allowing direct comparison of their contributions to biological activity.^31^ The lower potency generally observed for lysine-type relative to arginine-type peptoids has been attributed to the weaker membrane affinity of the primary ammonium group compared with the guanidinium group, which can engage in bidentate hydrogen bonding with phosphate headgroups.^31,32^ Furthermore, Amirkhanov and colleagues demonstrated that replacing arginine residues with lysine or histidine in synthetic antimicrobial peptides (SAMPs) led to a stepwise reduction in antibacterial activity, following the trend P1-Arg > P2-Lys > P3-His.^34^

### 3.2 Hydrophobicity

**Fig. 7:**
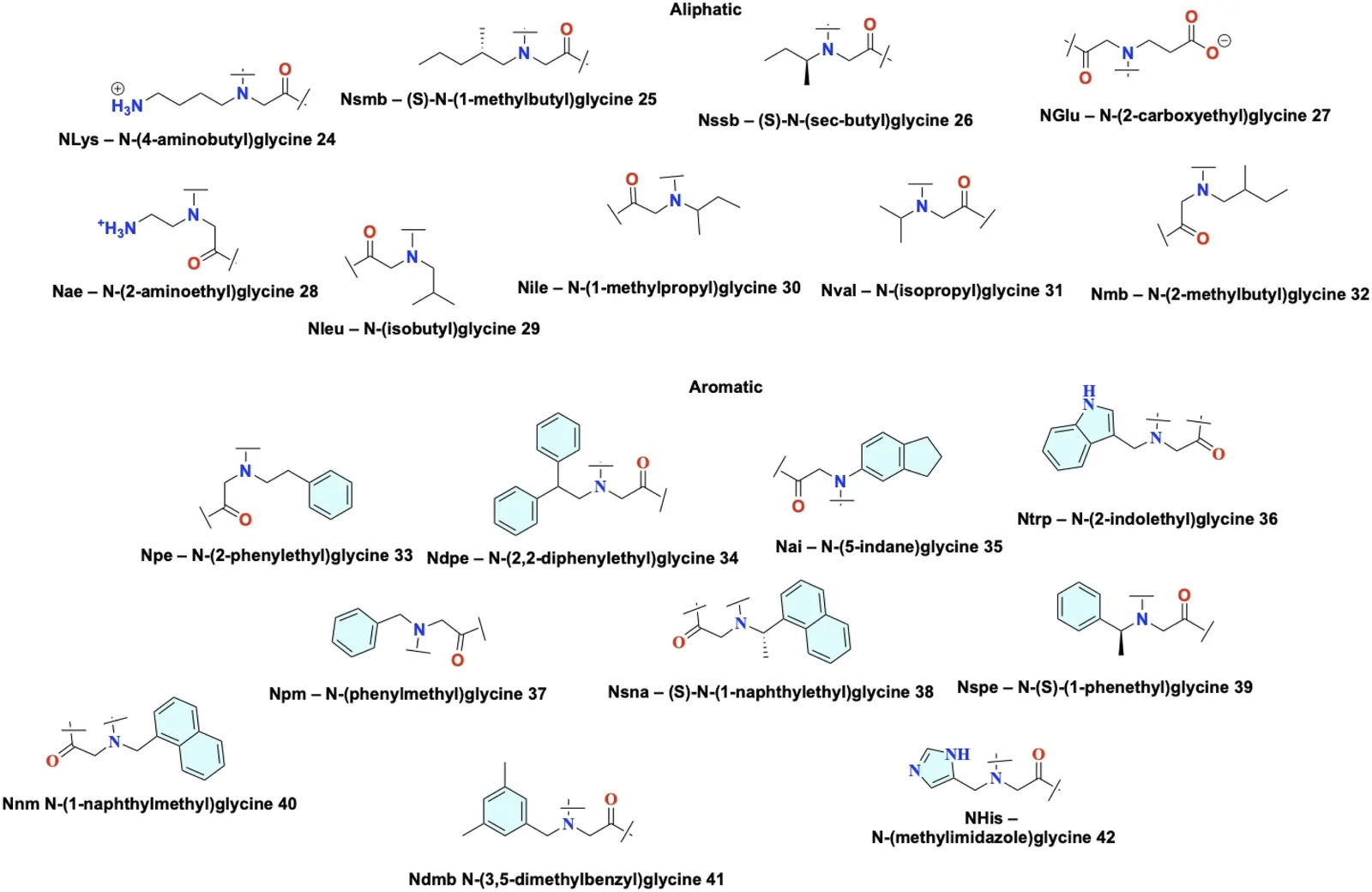
Aliphatic and aromatic side-chain derivatives used to modulate hydrophobicity.

Hydrophobic side chains are essential for membrane activity but must be tuned to avoid excessive toxicity. Attaching long alkyl tails to peptoids has been shown to significantly boost potency and selectivity:^35^ for instance, peptoids with 5–13 carbon tails often exhibited much higher antimicrobial activity and maintained selectivity compared to non-alkylated analogues. In one case, a tail at the N-terminus greatly enhanced potency against *Mycobacterium tuberculosis* by strengthening lipophilic membrane interactions.^36^ Another example is where the researcher investigated the effect of hydrophobicity by varying the inner alkyl linker length and the length of the terminal alkyl chain of the dimeric quaternary ammonium compounds on their activity against Gram-positive and Gram-negative bacteria.^37^ However, overly hydrophobic constructs tend to self-assemble (micellize) at higher concentrations, which can actually reduce effective membrane targeting. Moreover, the nature of the hydrophobic residue matters: aromatic side chains (*e.g.* indole or phenyl mimics) can rigidify the backbone and stack with lipids but may also decrease bacterial *vs.* host selectivity. In fact, the introduction of bulky aromatic residues has been observed to reduce the discrimination between bacterial and mammalian membranes.^38^ Careful side-chain choice mixing linear alkyl groups with selected aromatics is therefore used to fine-tune hydrophobicity and preserve amphipathic balance (Fig. 7).

The influence of hydrophobicity on peptoid activity has been demonstrated in a systematic structure–activity relationship study in which hydrophobicity was independently modulated without altering chain length. In the parent sequences H-(NLys-Nspe-Nspe)_4_-NH_2_ and H-(NLys-Nssb-Nspe)_4_-NH_2_, the Phe-like Nspe and Ile-like Nssb residues were replaced with the bulkier, more hydrophobic Nsna and Nsmb monomers^39^ (Fig. 7). These substitutions increased overall hydrophobicity and, for 2-Nsmb and 2-Nsna, led to enhanced antibacterial activity accompanied by increased hemolysis.

In contrast, replacement of Nspe with Nsna in an alternative sequence did not improve antibacterial potency but significantly increased haemolytic activity, highlighting the strong dependence of biological outcomes on both hydrophobicity and sequence context.^39^ The effects of reduced hydrophobicity were also examined. Substitution of two evenly spaced Nspe residues with the polar, weakly charged histidine analogue NHis produced 1-NHis, which showed a substantial decrease in haemolytic activity and cytotoxicity while retaining antibacterial activity against *Bacillus subtilis*.^40^

### 3.3 Amphiphilicity as a design principle

Most potent membrane-active AMPs adopt amphipathic architectures in which hydrophobic and cationic residues segregate onto distinct faces of a helix or related secondary structure. Antimicrobial peptoids are similarly designed to be amphipathic (compound 43), combining hydrophobic side chains with positively charged residues. This structural feature promotes the initial electrostatic attraction between cationic residues and negatively charged bacterial phospholipid head groups, followed by hydrophobic interactions with the lipid acyl chains.^41^ Such amphipathicity underpins bacterial selectivity, reduces mammalian cytotoxicity, and enhances membrane permeabilization and molecular transport activity.

To probe how amphipathicity and charge distribution govern activity, isomeric peptoid analogues identical in composition but differing in monomer sequence were synthesised. Hoque and his colleagues showed that increasing alkyl side-chain length, and thus amphipathicity, broadened antimicrobial spectra and improved biofilm disruption while preserving low hemolysis.^33^ These findings indicate that optimised amphipathicity enhances both planktonic and biofilm activity. Another study demonstrated that redistribution of terminal charge increased the apparent hydrophobicity of peptoids. This modification produced minimal changes in activity against *S. aureus* but resulted in a four-fold increase in potency against Gram-negative bacteria, including *Pseudomonas aeruginosa* and *Escherichia coli*, with little effect on haemolytic activity.^20^

These observations are consistent with Wimley's model, which proposes that imperfect segregation of hydrophobic and charged residues enhances membrane-disruptive potency.^42^ Supporting evidence from peptide systems reinforces this concept. Perturbation of the hydrophobic face of melittin enhances pore-forming activity, while disruption of dimerization in the amphipathic peptide V13KL by introduction of a charged lysine residue into the hydrophobic face reduces mammalian toxicity.^43^ In this case, dimerization was proposed to facilitate access to mammalian membrane interfaces without productive membrane permeation.

**Fig. 8:**
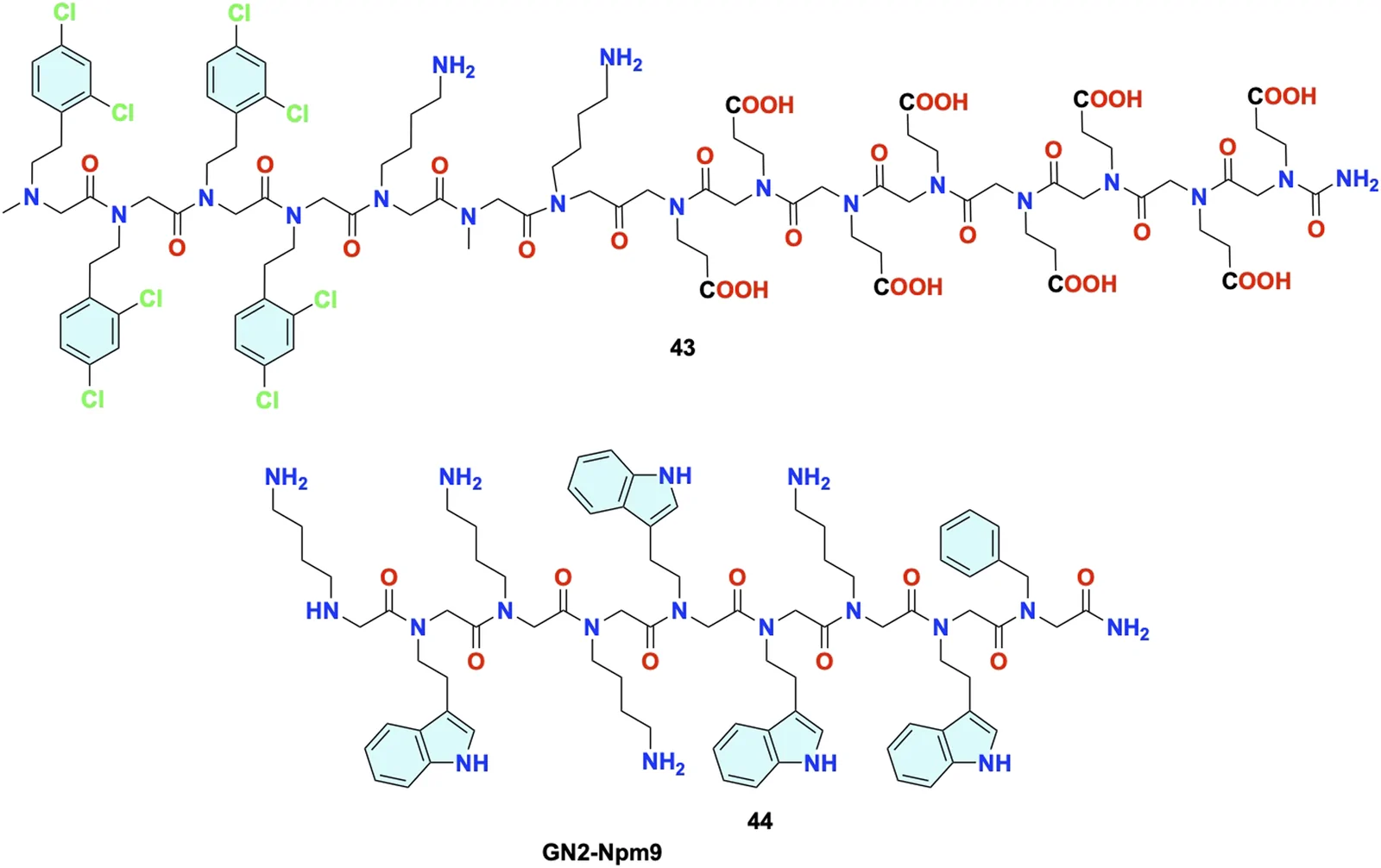
Representative amphiphilic peptoid architectures.

In another study, the synthetic peptoid GN2-Npm9 44 was engineered to exhibit enhanced amphiphilicity and was compared with other synthesized analogues^44^ (Fig. 8). The study demonstrated sustained release of the peptoid for at least nine days, indicating its potential for prolonged antimicrobial activity. The antibiofilm efficacy of GN2-Npm9 44 was further evaluated by functionalizing a nanometric, chemically pre-treated (CT) titanium surface intended for bone-contact implant applications. Multispecies bacterial biofilms, derived from clinical dental plaque, were directly inoculated onto the sample surfaces to assess activity.^44^

## 4 Architectures of antibacterial peptoids

### 4.1 Linear antibacterial peptoids

One of the most extensively explored SAR variables in antimicrobial peptoids is molecular topology, particularly the comparison between linear and cyclic architectures. Linear peptoids are synthetically straightforward and allow facile modulation of chain length, side-chain composition, and charge distribution.^1^ Early work by Barron and co-workers demonstrated that linear peptoids designed to adopt amphipathic conformations could effectively disrupt bacterial membranes, exhibiting activity comparable to natural AMPs against both Gram-positive and Gram-negative organisms.^45,46^

In 2003, Barron's group reported the first antibacterial Magainin-2 amide peptoid mimics, considered the earliest design of antibacterial peptoids.^45,47^ They designed 7 peptoids (12_mer_ to 17_mer_) using a repetitive sequence of *N*Lys, *N*ssp, and *N*spe monomers and investigated their antibacterial activity and selectivity. The most active compound against Gram-negative (*E. coli*) and Gram-positive bacteria (*B. subtilis*) was compound [H-(*N*Lys-*N*spe-*N*spe)_4_-NH_2_] peptoid 1 8 (12_mer_) (Fig. 5) with MICs of 6.3 µg mL^−1^ and 1.6 µg mL^−1^, respectively (44),^39^ The selectivity of the seven peptoids was determined by haemolytic activity; the most active molecule [H-(*N*Lys-*N*spe-*N*spe)_4_-NH_2_] was non-haemolytic at concentrations below 10 µg mL^−1^ while at higher concentrations it became haemolytic.^45^

Building on this framework, Zuckermann and Barron expanded the chemical diversity of linear peptoids by systematically varying hydrophobicity, charge, chirality, and chain length. In another study they selected two peptoid structures, ([H-(*N*Lys-*N*spe-*N*spe)_4_-NH_2_] peptoid 1 8 and [H-(*N*Lys-*N*ssb-*N*spe)_4_-NH_2_]) to design 13 new peptoid analogues that mimic known synthetic antimicrobial peptides, including pexiganan and melittin.^39^

In another example, the insertion of the *N*His chain in place of *N*spe generated a peptoid with a small improvement in activity against *B. subtilis* and a significant reduction in cytotoxicity and hemolysis *in vitro*. Also, *N*spe at position 6 in [H-(*N*Lys-*N*spe-*N*spe)_4_-NH_2_] peptoid 1 8 was replaced with _L_Pro to give peptoid H-*N*Lys-*N*spe-*N*spe-*N*Lys-*N*spe-_L_Pro-(*N*Lys-*N*spe-*N*spe)_2_-NH_2_ with less hemolysis and cytotoxicity.^39^

Another linear peptoid was designed in 2018 by the groups of Seo and Barron to enhance the activity and selectivity of peptoid 1 (5) against *Escherichia coli* and *Bacillus subtilis*. Structural modifications involved the incorporation of additional hydrophobic and lysine residues. The optimized peptoid, H-(Nlys-Nspe-Nspe)_3_-Nlys-NH_2_1 (Fig. 5), exhibited increased antibacterial activity, with MIC values of 2.4 µg mL^−1^ against *E. coli* and 1.2 µg mL^−1^ against *B. subtilis*, while showing reduced cytotoxicity relative to the parent compound peptoid 1 8.^48^

**Fig. 9:**
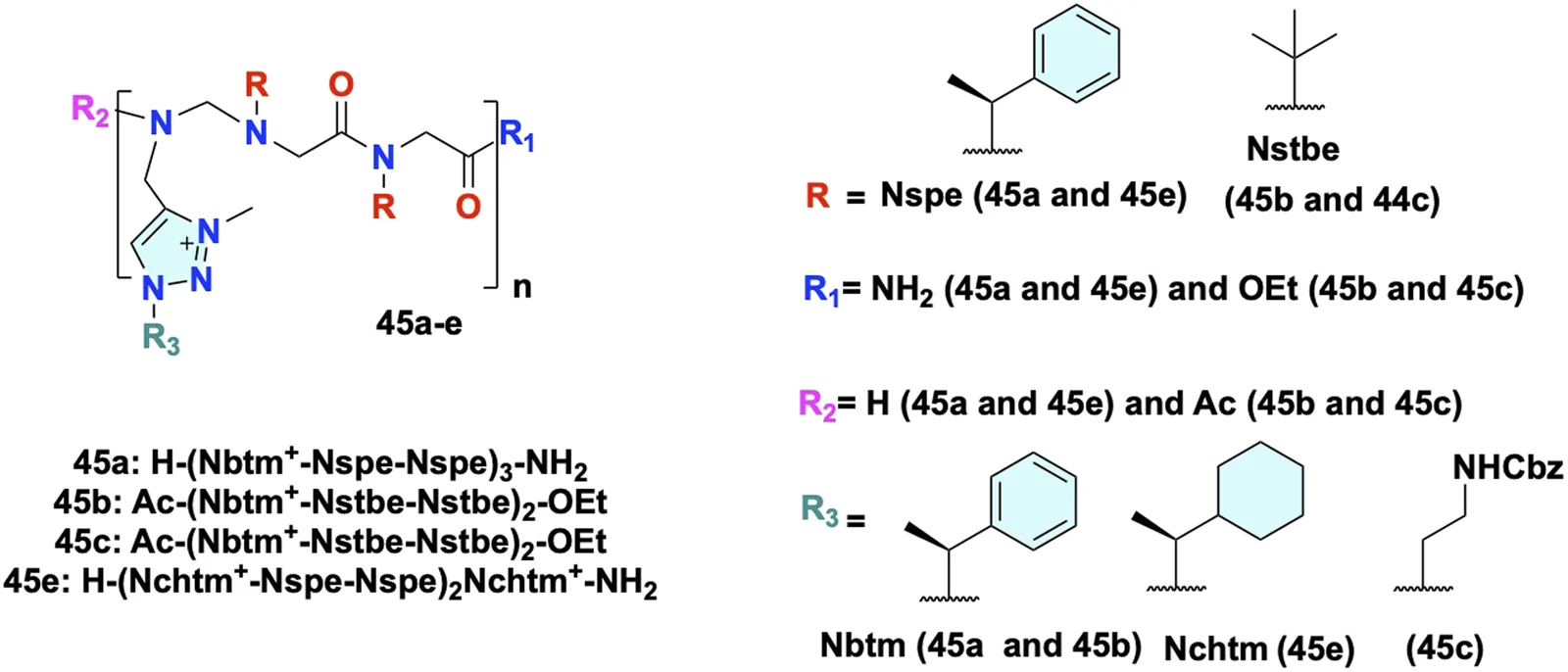
Highly potent antimicrobial peptoids developed across various studies by the Faure group. Nspe refers to (*S*)-*N*-(1-phenylethyl)glycine; Nbtm to *N*-(1-benzyl-1,2,3-triazolylmethyl)glycine; and Nchtm to *N*-(1-cyclohexylmethyl-1,2,3-triazolylmethyl)glycine. The cationic derivatives include Nbtm^+^, defined as *N*-(1-benzyl-3-methyl-1,2,3-triazolium methyl)glycine, and Nchtm^+^, defined as *N*-(1-cyclohexylmethyl-3-methyl-1,2,3-triazolium methyl)glycine.

**Fig. 10:**
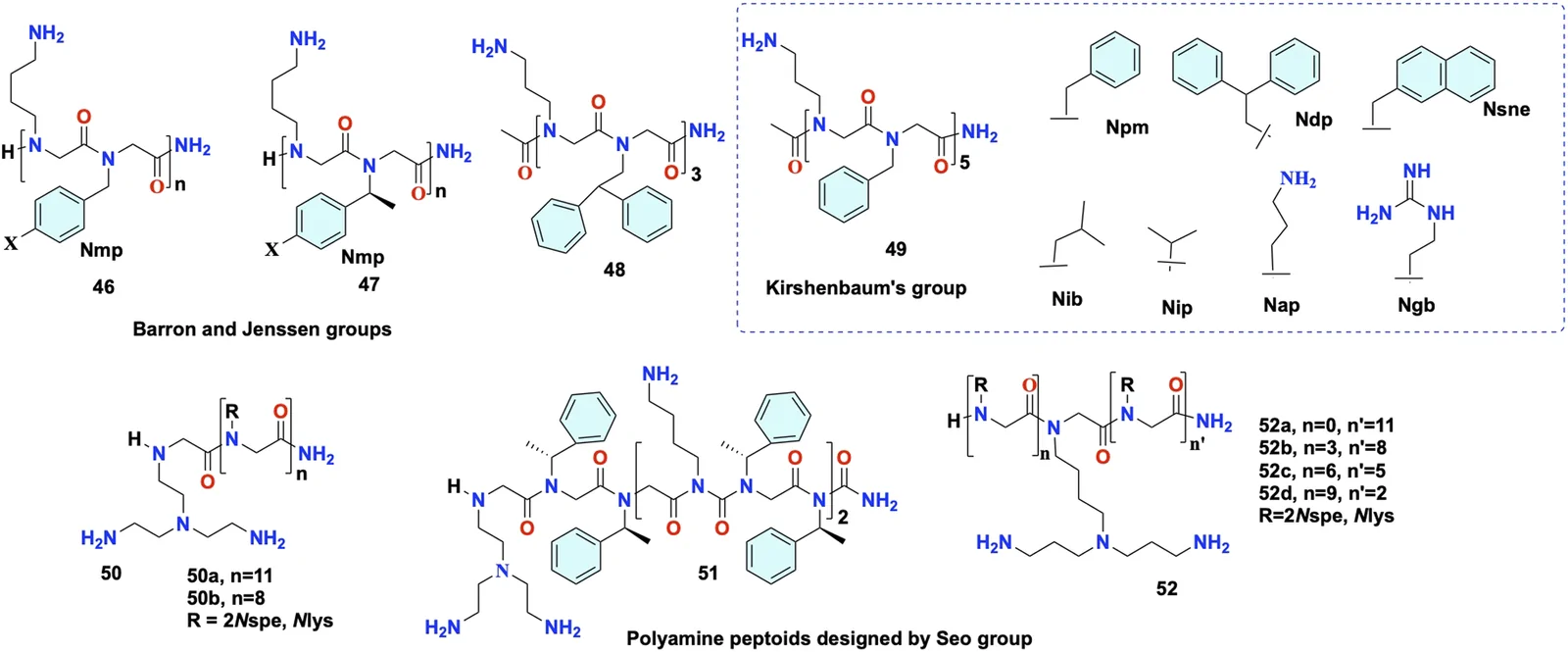
Active linear antibacterial peptoids designed by different research groups.

In 2018, the Faure group replaced *N*Lys part in peptoid 1 (8) structure (Barron group 2003) with triazolium to investigate the role of cationicity of 1,2,3-triazolium on the activity against Gram-negative and Gram-positive bacteria.^49^ The most active peptoid against *S. aureus* was H-(*N*btm^+^-*N*spe-*N*spe)_3_-NH_2_45a (Fig. 9) with MIC of 3.3 µg mL^−1^, and the most active peptoids against *E. coli* were H-(*N*btm^+^-*N*spe-*N*spe)_3_-NH_2_45a and H-(*N*aetm-*N*spe-*N*spe)_4_-NH_2_ with MIC of 12.8 µg mL^−1^ and were nontoxic with HC_50_ > 200. Additionally, in the next study, they designed trimeric and hexameric 1,2,3-triazolium peptoids and investigated their antibacterial activity against a wide range of Gram-positive and Gram-negative bacteria. Notably, hexameric peptoids 45b and 45c proved to be the most potent^50^ (Fig. 10). Recently, the Faure group proved the ability of 1,2,3-triazolium amphipathic peptoids to damage the bacterial cell membrane *via* selective disruption by changing the nature of the triazolium group by attaching it with additional hydrophobic or lipophilic groups, and cationic residues by varying the length of the sequence and the 7_mer_ peptoid 45e was the most effective molecule (Fig. 10).^51^

In 2019, the Cobb group demonstrated that fluorinated aromatic monomers can modulate the *cis*/*trans* equilibrium of α-peptoids, with electron-withdrawing fluorinated substituents significantly enhancing *cis*-amide preference and enabling conformational control without the need for chiral side chains^52^ In 2020, Barron and Jenssen groups designed 36 peptoids utilizing *p*-halogenated phenyl with (*N*Lys, *N*pm) or (*N*Lys, *N*spe)_*n*_ units (Fig. 10).^53^ Activity depended on the identity of the halogen, the degree of substitution and the chain length, and was measured against seven strains: five Gram-positive (*S. aureus* ATCC 25923 and ATCC 29213, methicillin-resistant *S. aureus* USA300, and methicillin-resistant S. epidermidis ET-024 and ATCC 51625) and two Gram-negative (*E. coli* ATCC 25922 and *P. aeruginosa* PAO1). Incorporation of chlorine or bromine into the (NLys–Npm)_*n*_ scaffold enhanced activity against Gram-positive bacteria, whereas fluorination had no pronounced effect. For the 6- and 8-mers, potency against Gram-positive strains rose in the order F < Cl < Br < I, the fully iodinated 6-mer showing a greater than 64-fold improvement against *S. aureus* and a 256-fold improvement against methicillin-resistant *S. epidermidis* relative to the non-halogenated control. Importantly, halogenation of this first-generation library had no measurable effect on activity against *E. coli* or *P. aeruginosa*, indicating that the benefit was confined to Gram-positive organisms in this scaffold. The most active compounds were H-(*N*lys-*N*mp(*p*-Cl))_5or6_-NH_2_55 (Table 1) with MICs of 2 µg mL^−1^ against *S. aureus* and 64 µg mL^−1^ against *E. coli*. For the 10- and 12-mers the halogen trend broke down: the fully iodinated analogues lost activity, which was attributed to hydrophobicity exceeding a threshold and driving self-assembly into discrete bundles. Bromination of the 10-mer, by contrast, produced a greater than 256-fold increase in activity against *S. aureus* and a 128-fold increase against methicillin-resistant *S. epidermidis* relative to the unsubstituted control. Introducing chlorine and bromine to (*N*Lys–*N*spe) resulted in a dramatic decrease in efficacy.^53^ In a separate study, the most active compounds against *E. coli* were (*N*hArg*N*phe*N*phe)_4_, [(*N*Lys*N*pfb*N*pfb)(*N*Lys*N*spe*N*spe)]_2_, (*N*hArg*N*spe*N*spe)_4_.^54^ (Table 1), (*N*hArg*N*spe*N*spe)_3_, and (*N*hArg*N*mfb*N*mfb)_3_ with MIC of 6 µg mL^−1^, while the most active compounds against *P. aeruginosa* were (*N*Lys*N*pfb*N*spe)_4_, (*N*hArg*N*spe*N*spe)_3_ and (*N*hArg*N*mfb*N*mfb)_4_ with MIC of 13 µg mL^−1^.

**Table 1:** Selected examples of the most active linear antibacterial peptoids with their MICs

Peptoid structure	MIC	Ref.
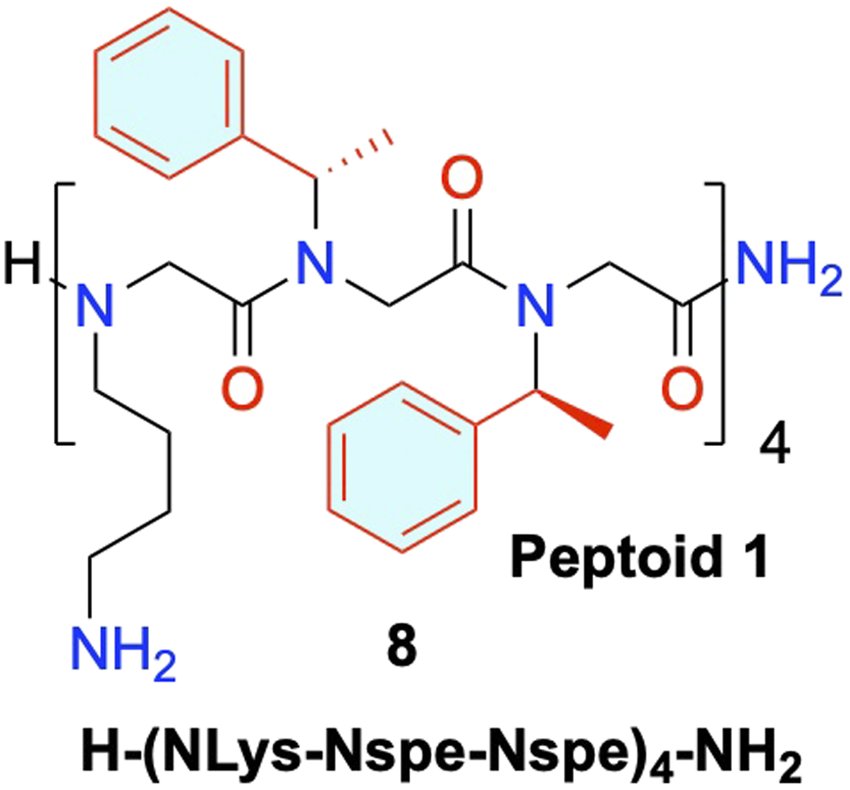	1.56 µg mL^−1^/*S. aureus*	39 and 55
	6.3 µg mL^−1^/*E. coli*	
	1.6 µg mL^−1^/*B. subtilis*	
	12.5 µg mL^−1^/*P. aeruginosa*	
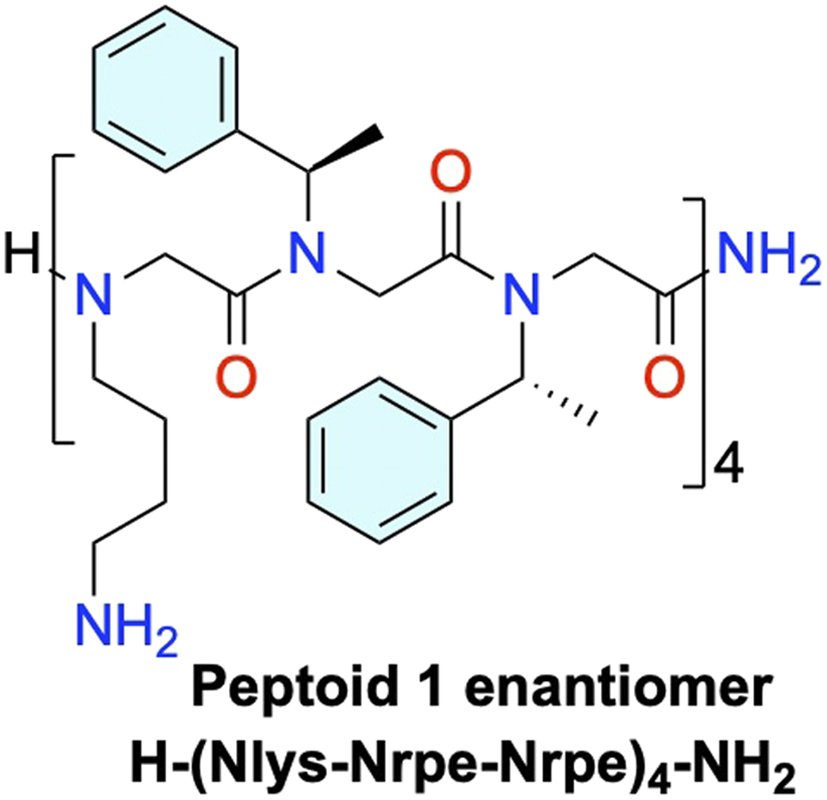	6.3 µg mL^−1^/*E. coli*	39
	1.6 µg mL^−1^/*B. subtilis*	
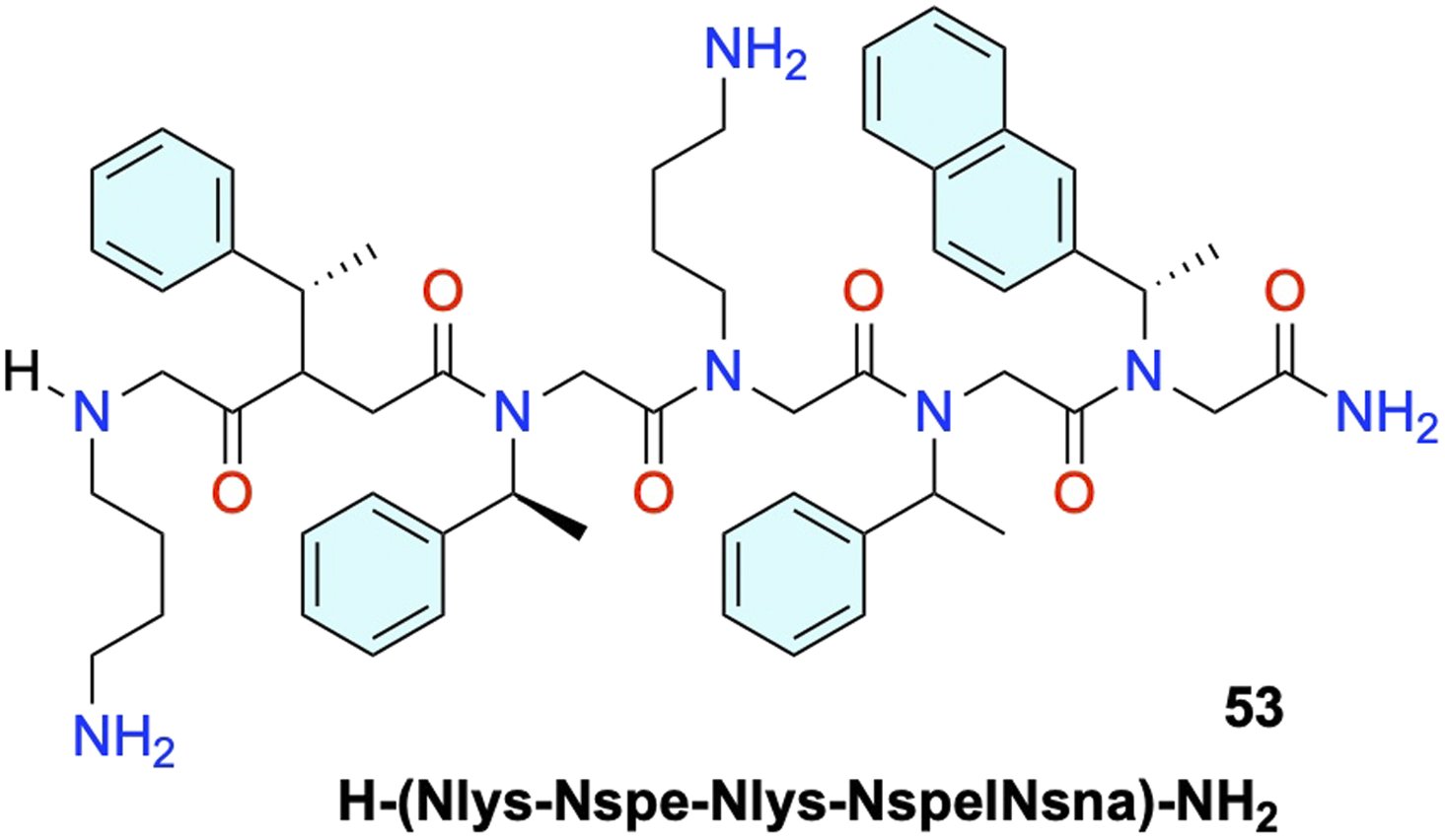	3.2 µg mL^−1^/*E. coli*	39
	1.5 µg mL^−1^/*B. subtilis*	
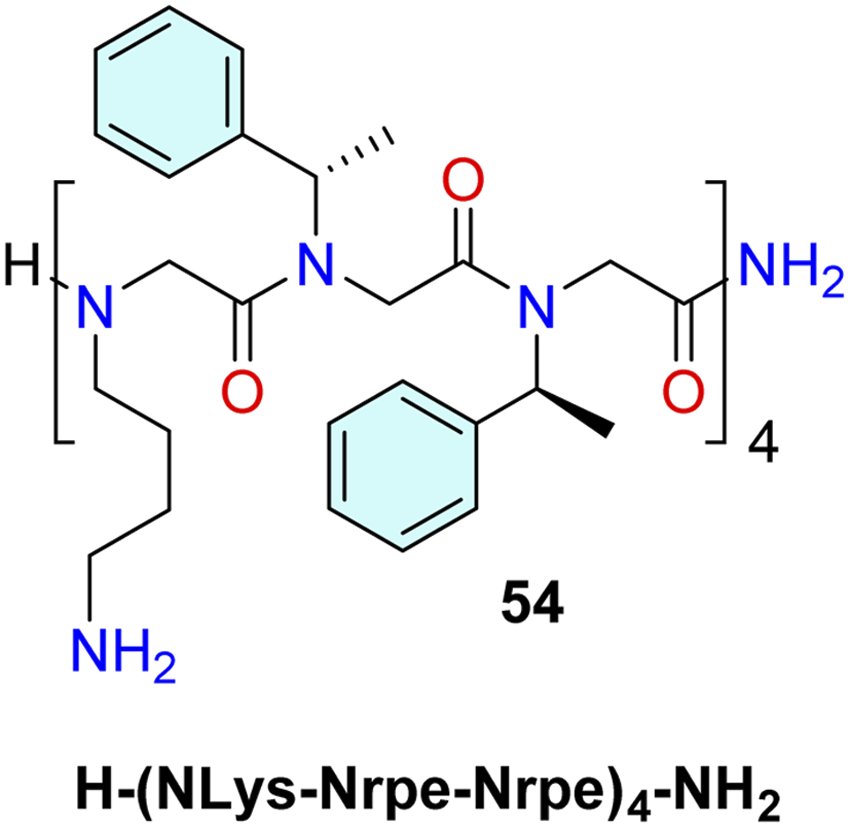	2 µg mL^−1^/*S. aureus*	54
	6 µg mL^−1^/*E. coli*	
	13 µg mL^−1^/*P. aeruginosa*	
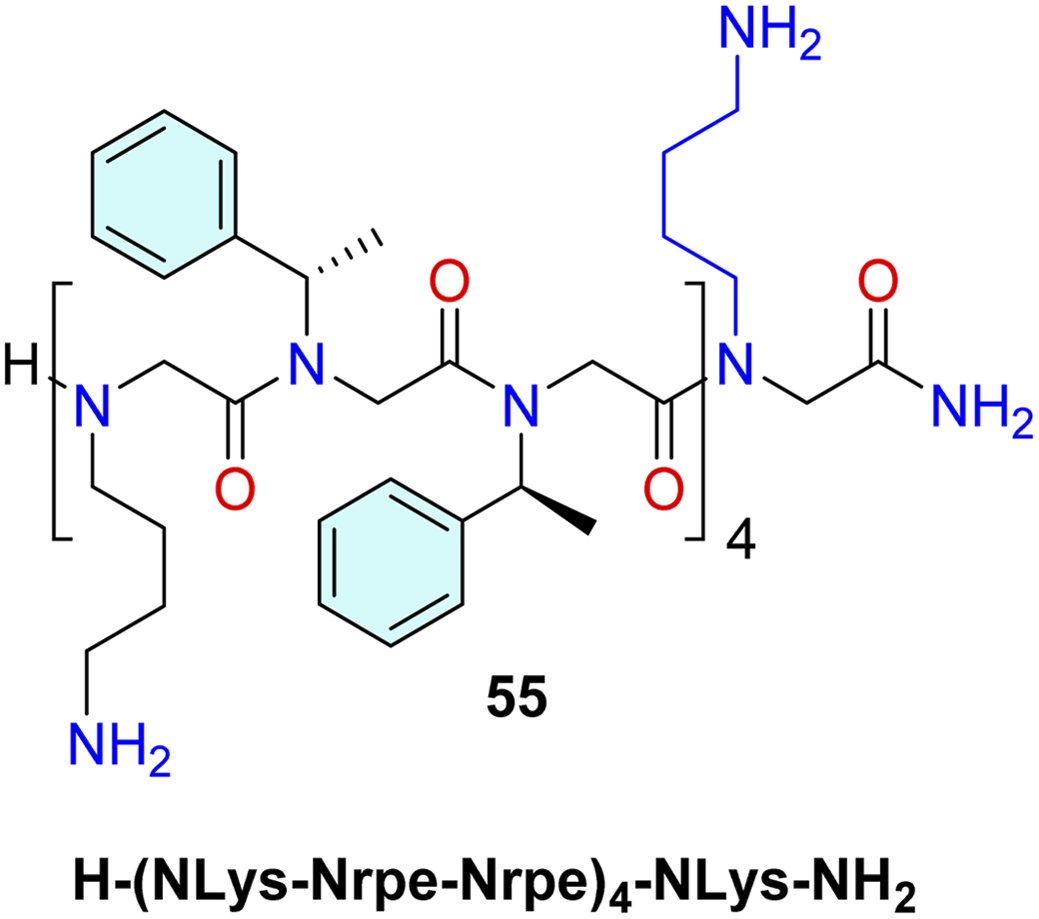	1.2 µg mL^−1^/*B. subtilis*	48
	2.4 µg mL^−1^/*E. coli*	
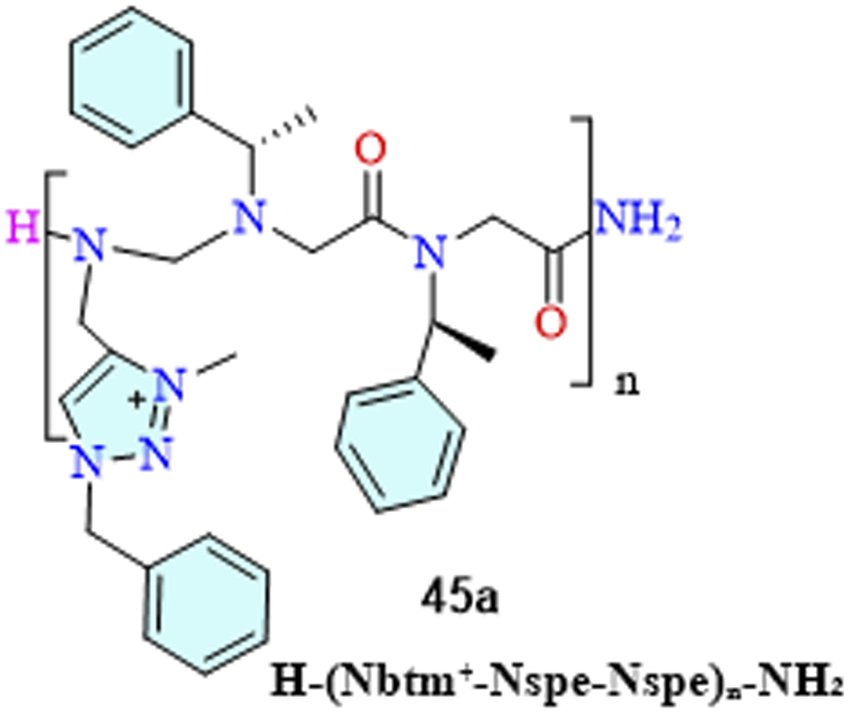	3.3 µg mL^−1^/*S. aureus*	49
	12.8–25.4 µg mL^−1^/*E. coli*	
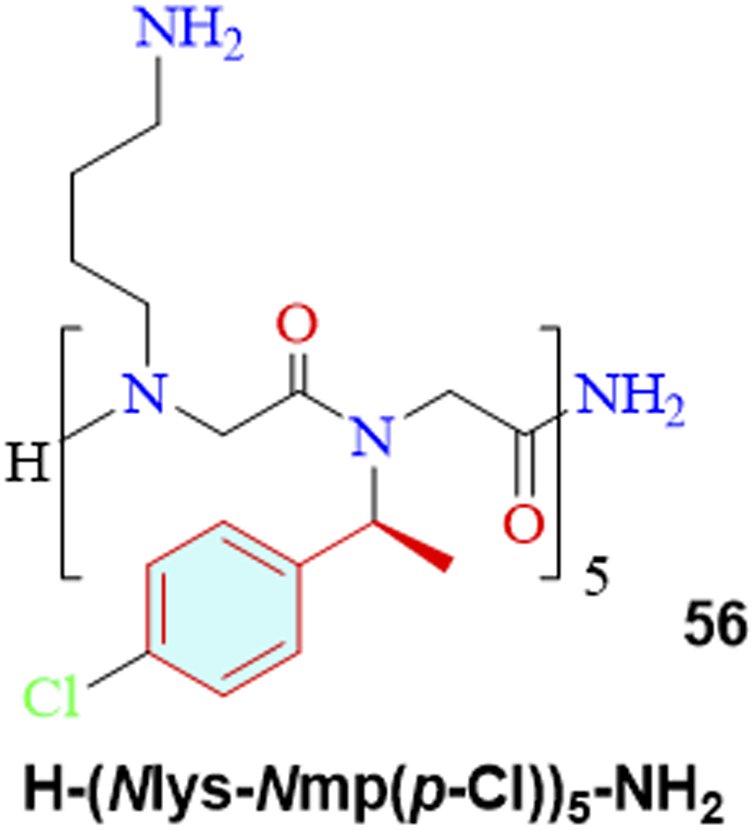	2 µg mL^−1^/*S. aureus*	53
	64 µg mL^−1^/*E. coli*	
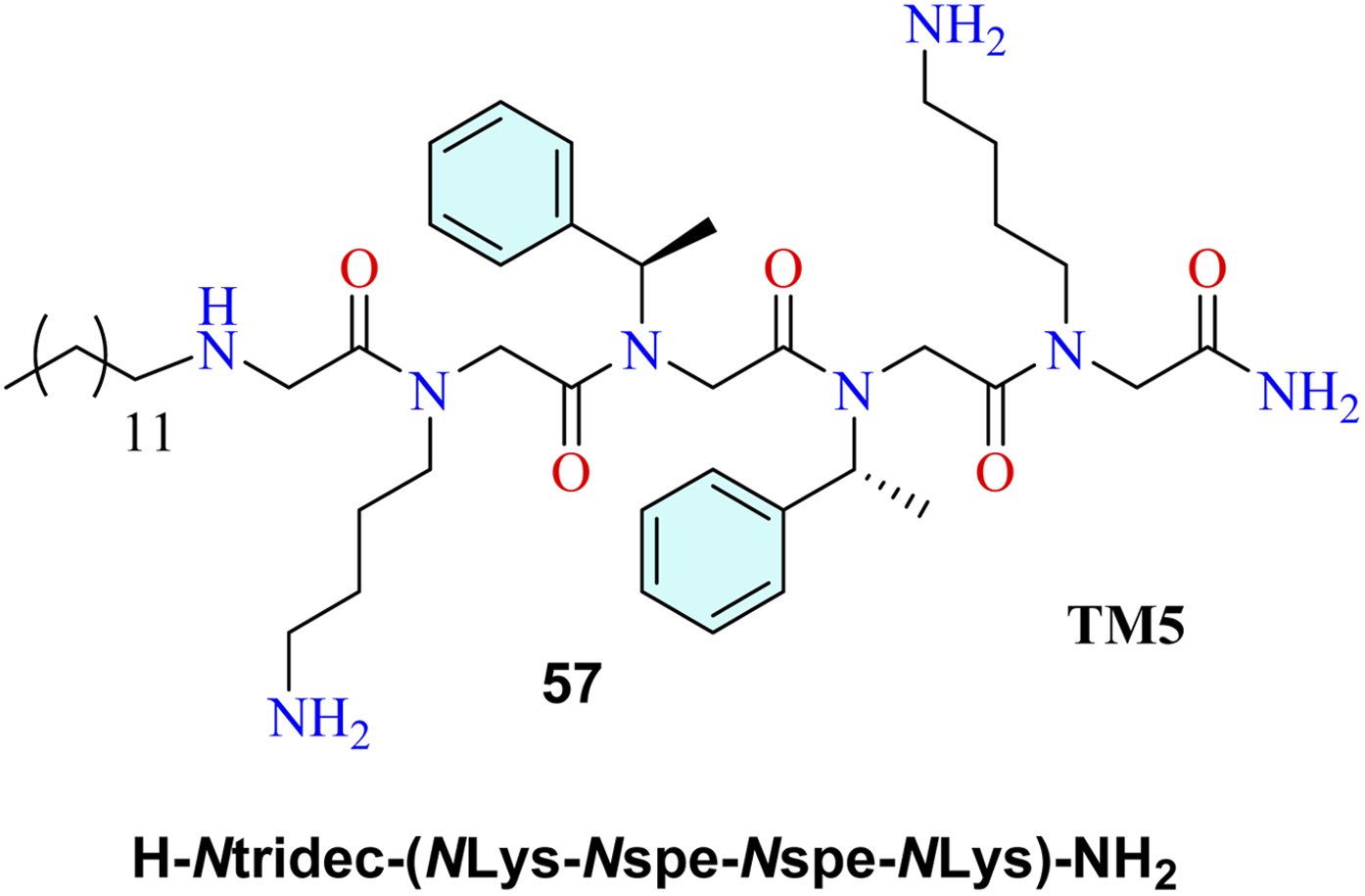	1.56 µg mL^−1^/*S. aureus*	35 and 55
	3.13 µg mL^−1^/*P. aeruginosa*	
	1.8 µg mL^−1^/*B. subtilis*	
	14 µg mL^−1^/*E. coli*	

Additionally, Jenssen's group studied various cationic amphipathic (9_mer_) peptoid residues with +4 net charge and various hydrophobic groups (aromatic and aliphatic side chains), as well as those with changes in the cationic residue and its position. They designed 22 cationic peptoids and reported that the high hydrophobicity increases the activity against *S. aureus* but does not play a role against Gram-negative bacterial strains. The best aromatic sub-monomer units were *N*-(2-indolethyl) glycine (*N*trp) and *N*-(5-indane) glycine (*N*ai). On the other hand, the introduction of aromatic side chains with the highest hydrophobicity, such as the *N*-(2,2-diphenyl- ethyl) glycine (*N*dpe) group (Fig. 9), caused conjugation with a loss of bacterial activity and increased toxicity.^20^

**Fig. 11:**
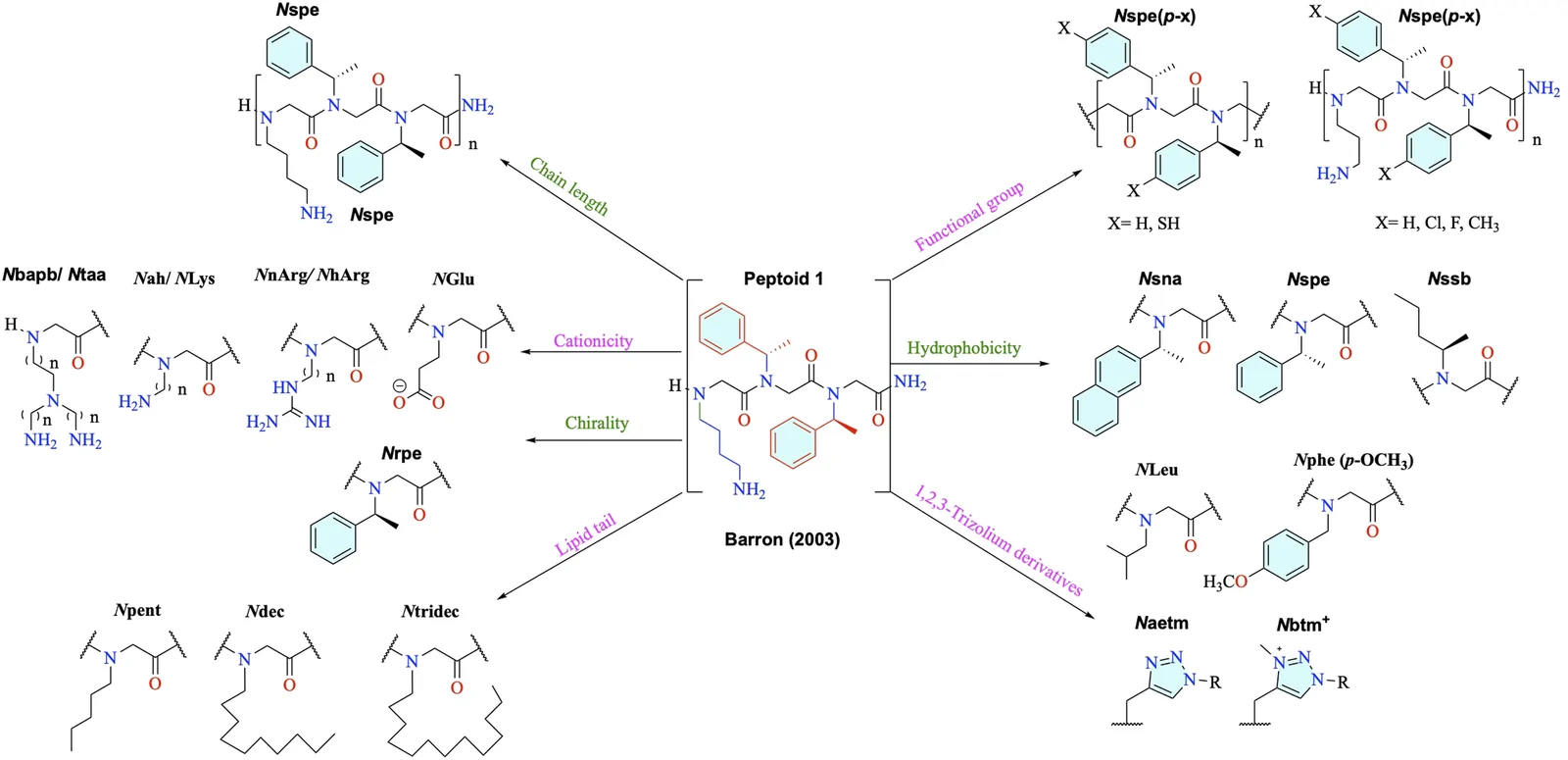
Summary of the modification of peptoid 1 structure.

Recent study has also inserted new cationic monomers, including *N*taa and *N*bapb, alongside the common monomers *N*spe and *N*Lys to different peptoids backbones, including Barron group's peptoid 1 8 and other analogs. They connected an additional alkylated cationic group to the lysine residue of peptoid 1 8 and some of its derivatives 50a and 52. Peptoid 50b H-(*N*taa-*N*spe-*N*spe)-(*N*Lys-*N*spe-*N*spe)_2_-NH_2_ showed an enhancement in the antibacterial selectivity against *S. aureus* and against *E. coli*, as well as a significant reduction in the toxicity and bacterial resistance compared to the parent peptoid 1 8 (Fig. 10).^56^Fig. 11 shows a diagram that summarizes the structure modification of peptoid 1 8 that was first reported by the Barron group.

Kirshenbaum's group employed different cationic and hydrophobic residues, including *N*ap, *N*ab, *N*ah, *N*gb, *N*pm, *N*nm, *N*dp, *N*ip, and *N*ib to design linear cationic peptoids of six to ten residues (three to five repeating cationic–hydrophobic dimers) with moderate activity against *S. aureus*, *B. subtilis*, and *E. coli*. Peptoid 48 Ac-(*N*apNdp)_3_-NH_2_ (Fig. 10) was the best active linear peptoid against *S. aureus*, *B. subtilis*, and *E. coli* with MICs of 15.6 µg mL^−1^, 1 µg mL^−1^, and 15.6 µg mL^−1^, respectively.^57^ Peptoid 49 Ac-(*N*ap*N*pm)_5_-NH_2_ (Fig. 10) was in the second order with MICs of 62.5 µg mL^−1^, 15.6 µg mL^−1^, and 31.3 µg mL^−1^ against *S. aureus*, *B. subtilis*, and *E.coli*, respectively.^57^

In a recent study, the Seo group inserted new cationic monomers, including *N*taa and *N*bapb, alongside the common monomers *N*spe and *N*Lys to different peptoid backbones. They connected an additional alkylated cationic group to the lysine residue of peptoid 1 8 and some of its derivatives 50 and 52. Peptoid 50b H-(*N*taa-*N*spe-*N*spe)-(*N*Lys-*N*spe-*N*spe)_2_-NH_2_ showed an enhancement in the antibacterial selectivity against *S. aureus* and against *E. coli*, as well as a significant reduction in the toxicity and bacterial resistance compared to the parent peptoid 1 8 (Fig. 11).

### 4.2 Lipopeptoids

**Fig. 12:**
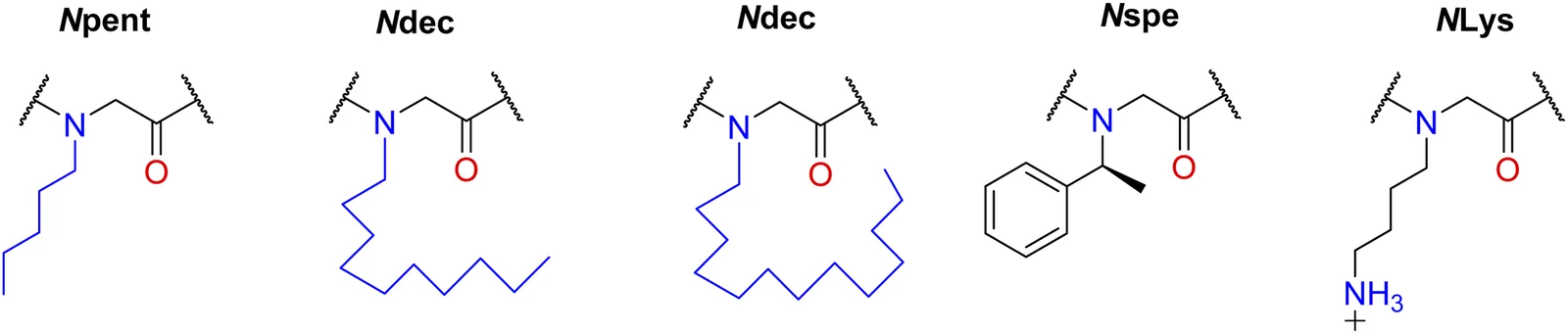
Lipopeptoid monomer side chain (Barron Group 2010).

The Barron group designed short lipopeptoid mimics of antimicrobial lipopeptides.^35^ Linear peptoids 8 from the Barron group were used as essential backbones and connected at the N-terminus to alkyl chains of 5, 10 or 13 carbons (lipid-type chains) (Fig. 12).

Lipidation length differentially affected antibacterial activity. Increasing the alkyl chain enhanced activity against Gram-negative bacteria but reduced potency against Gram-positive strains. For example, Npent-1 (C5) and Ndec-1 (C10) displayed MICs of 15.5 and 31.6 µg mL^−1^, respectively, against *E. coli*. In shorter 9mer analogues, the C5 variant showed MICs of 30.8 µg mL^−1^ (*E. coli*) and 1.8 µg mL^−1^ (*B. subtilis*), whereas the C10 analogue improved activity against *E. coli* (12.4 µg mL^−1^) but reduced potency against *B. subtilis* (6.2 µg mL^−1^)^35^ sequence length was also critical. The shortest 4mer bearing a C10 chain lost activity against *E. coli* (MIC >108 µg mL^−1^), while the 6mer with the same lipid chain showed the highest potency (MIC 8.8 µg mL^−1^), indicating an optimal balance between lipid length and backbone size.^58,59^

The best lipopeptoid against Gram-positive (*B. subtilis*) was *N*tridec-1_4mer_ (H-*N*tridec-(*N*Lys-*N*spe-*N*spe-*N*Lys)-NH_2_) (TM5) 57 (Table 1) with MIC of 1.8 µg mL^−1^, which is the shortest peptoid with the longest side chain with 13C. Further analysis also shows that more hydrophobic compounds exhibited the highest toxicity; however, the alkyl residue was not the only factor contributing to this toxicity.^35^

The authors further assessed lipidated analogues against ESKAPE pathogens, including the 4mer H-Ndec-(NLys-Nspe-Nspe-NLys)-NH_2_, the 6mer (H-Ndec-(NLys-Nspe-Nspe)_2_-NH_2_), and the *p*-brominated 6mers with C10 or C13 chains, benchmarking them against parent peptoid 1 8.^60^ Against *S. aureus*, the most potent compounds were peptoid 1 8, the C13-lipidated 4mer H-Ntridec-(NLys-Nspe-Nspe-NLys)-NH_2_, and the brominated 6mer C10 analogue (H-Ndec-(NLys-Nspe-Nspe(p-Br))_2_-NH_2_), each showing MIC = 1.56 µg mL^−1^. Against *P. aeruginosa*, TM5 (57); H-Ntridec-(NLys-Nspe-Nspe-NLys)-NH_2_) was most active, with MIC = 3.13 µg mL^−1^, highlighting the benefit of longer lipid tails and aromatic bromination for Gram-negative antibacterial activity.^60^

**Fig. 13:**
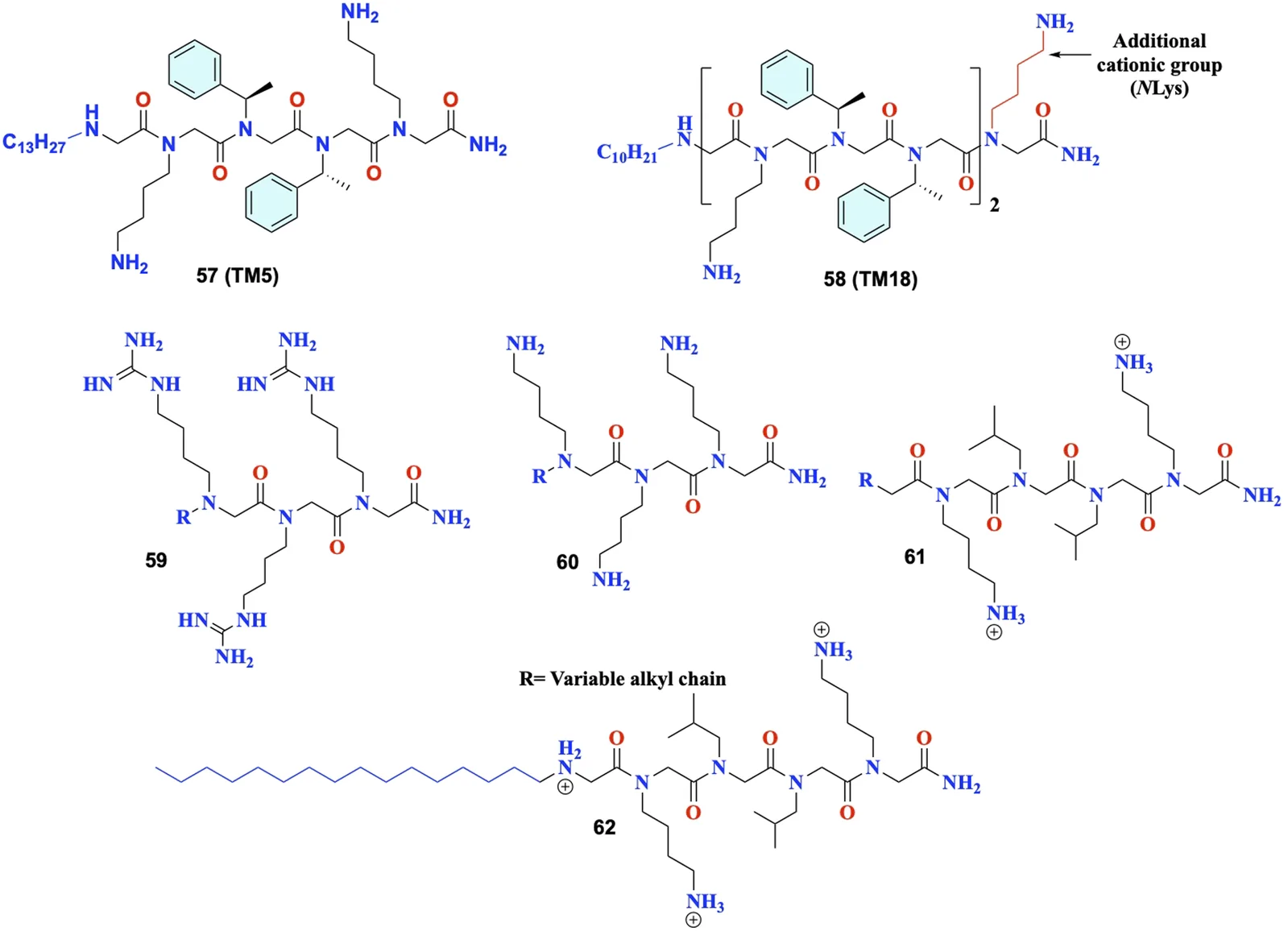
Examples of active lipopeptoids.

Additionally, in a new study, the Barron group added the *N*lys group to the C-terminal of their previously designed lipopeptoids, and they report that the modified peptoid (TM18) (H-*N*dec-(*N*Lys-*N*spe-*N*spe)_2_-*N*Lys-NH_2_) 58 (Fig. 13) was the potent peptoid with MIC of 6.3 µg mL^−1^ against *P. aeruginosa*.^61^

A novel library of 19 very short guanidino and amino lipopeptoids 59 and 60 (Fig. 13) was synthesized by the Schweizer group.^62^ The activity of these 19 compounds was investigated against a range of clinical Gram-positive and Gram-negative bacterial strains as per CLSI macro broth standards. Green and Bicker designed lipopeptoid mimics 61 (Fig. 13) and evaluated their activity against Gram-positive and Gram-negative bacterial strains. Increased hydrophobicity enhanced antimicrobial activity but could increase cytotoxicity. The most active compound, MG10 (62), showed an MIC of 6.3 µg mL^−1^ against *P. aeruginosa*.^63^

### 4.3 Cyclic peptoids

Cyclic peptoids have emerged as an important subclass of peptidomimetics due to their conformational rigidity, enhanced proteolytic stability, and potential for improved membrane selectivity compared with linear analogues.^64^ Conformational constraint through cyclization can promote defined amphipathic architectures, thereby strengthening interactions with bacterial membranes while modulating cytotoxicity.^65^

Over several years, Kirshenbaum's group systematically designed antibacterial cyclic peptoids incorporating diverse hydrophobic and cationic groups and evaluated their activity and selectivity.^41,57^ In their initial study, Huang *et al.* reported a library of linear and cyclic peptoids ranging from hexamers to decamers and directly compared their antimicrobial profiles.^57^

**Fig. 14:**
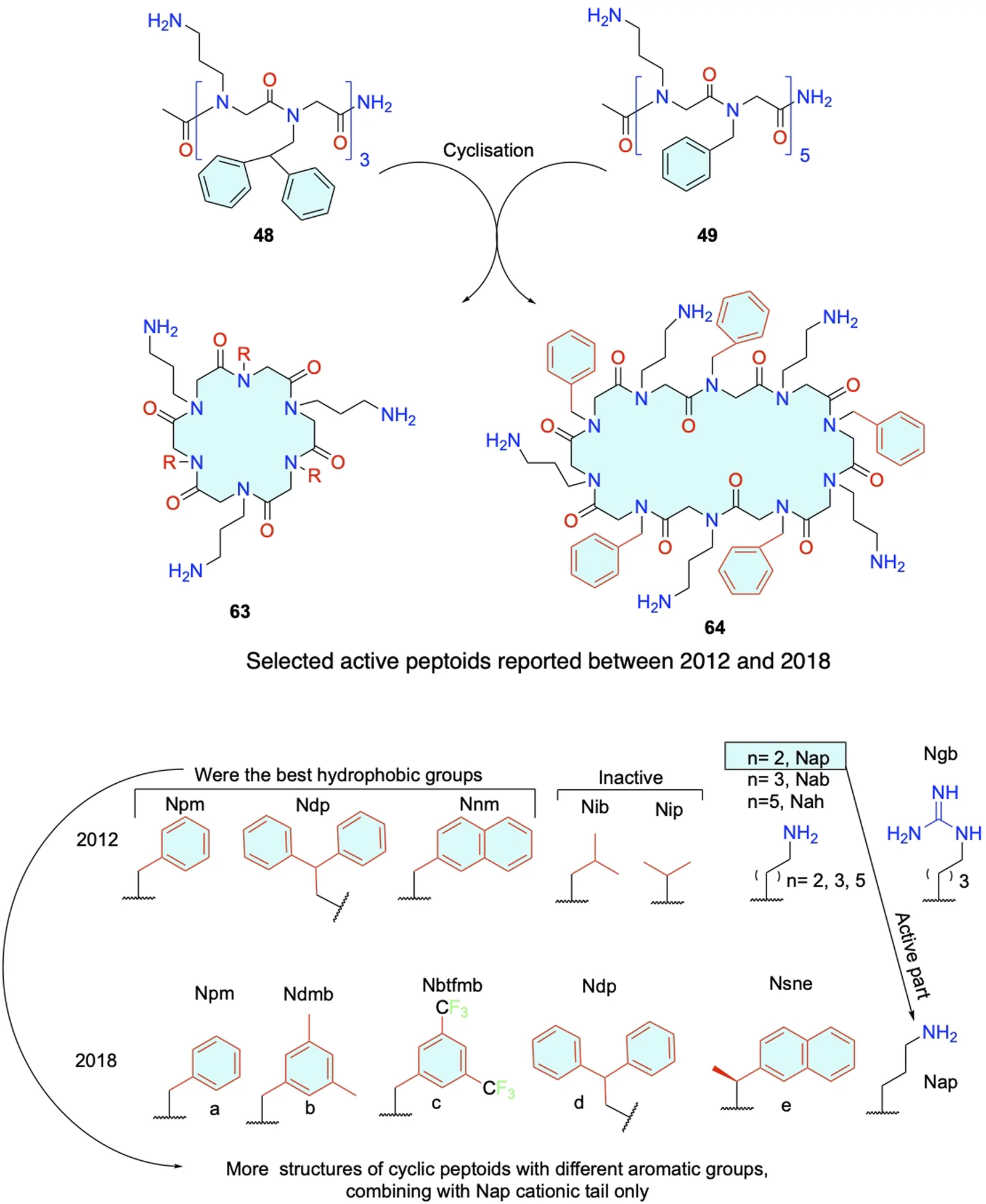
Active antibacterial cyclic peptoids designed by Kirshenbaum's group. *N*pm: *N*-(phenylmethyl)glycine; *N*nm: *N*-(1-naphthylmethyl)glycine; *N*dp: *N*-(2,2-diphenylethyl)glycine; *N*sne: (*S*)-*N*-(1-naphthylethyl)glycine; *N*ib: *N*-(isobutyl)glycine, *N*ip: *N*-(isopropyl)glycine; *N*dmb: *N*-(3,5-dimethylbenzyl)glycine; *N*btfmb: *N*-(3,5-bis-trifluoromethylbenzyl)glycine; *N*ap: *N*-(3-aminopropyl)glycine; *N*ab: *N*-(4-aminobutyl)glycine; *N*ah: *N*-(6-aminohexyl)glycine; *N*gb: *N*-(4-guanidinobutyl)glycine.

Within both series, the 6mer and 10mer 64 constructs showed the strongest activity against Gram-positive (*B. subtilis*, *S. aureus*) and Gram-negative (*E. coli*) bacteria. Cyclisation was generally, but not uniformly, favourable. The cyclic decamer 64 was the most potent member of the cyclic series against *E. coli* (MIC 7.8 µg mL^−1^), an eight-fold improvement over the corresponding linear decamer 49 (31.3 µg mL^−1^), whereas the cyclic hexamer 63 matched rather than exceeded its linear counterpart 48 against *B. subtilis* and *E. coli* (1 and 15.6 µg mL^−1^ for both). The most potent single value recorded in the cyclic series was 0.5 µg mL^−1^ against *B. subtilis*, obtained for a naphthylmethyl-containing cyclic hexamer. Individual values should be read from the primary report, since medium and inoculum differ between the studies compared here (Fig. 14).^57^

In subsequent work, the group investigated how hydrophobicity influences the selectivity of cyclic peptoids toward negatively charged bacterial membranes. They demonstrated that subtle modifications to hydrophobic side chains significantly alter membrane interactions and biological selectivity, establishing hydrophobic fine-tuning as a key design principle for potent and selective cyclic peptoids.^41^

## 5 Side-chain modifications

### 5.1 Aromatic side chains

Aromatic side chains are widely employed in antibacterial peptoids to increase hydrophobic surface area, promote membrane partitioning, and enable π–π and cation–π interactions that influence both activity and selectivity.^66,67^ Common aromatic monomers such as Npm, Nspe, Nnm and tryptophan-like (Ntrp-type) residues contribute substantial lipophilicity and can bias backbone conformation through steric and electronic effects, including modulation of amide *cis*–*trans* equilibria and backbone dihedral preferences.^66,68^ These conformational effects indirectly shape amphipathic presentation, which is critical for membrane disruption.

Systematic substitution studies by Mojsoska *et al.* demonstrated that replacing moderately hydrophobic aromatic residues with bulkier analogues in their reference compound GN-2, such as substituting Nspe with Ndpe or introducing Ntrp-type residues at selected positions, often enhanced activity against *S. aureus*. However, these gains were frequently accompanied by increased haemolysis and cytotoxicity toward HeLa cells.^20^ In several cases, substitution of a single aromatic residue resulted in a multi-fold reduction in MIC alongside a marked increase in haemolytic activity, highlighting the high sensitivity of peptoid structure–activity relationships to side chain identity.^20,69^

Despite this trade-off, certain aromatic monomers can achieve a more favorable balance between antibacterial potency and toxicity. Ntrp-type residues, when introduced in limited numbers and at appropriate positions, have been shown to enhance antibacterial activity without inducing excessive haemolysis.^20,70^ These indicate that fine-grained tuning of aromatic content and spatial distribution is a critical strategy for optimising peptoid performance. While hydrophobic side chains are essential for membrane activity, excessive hydrophobicity compromises selectivity and increases mammalian membrane disruption.

Aromatic side chains have also been utilized by Huang *et al.* to investigate the role of hydrophobic surface area using linear and macrocyclic peptoids of fixed length and charge but varying aromatic content. Peptoids containing three phenyl residues were compared with analogues bearing either three naphthyl residues or six phenyl residues, thereby spanning a wide range of hydrophobic surface areas. In both linear and macrocyclic series, increased aromatic surface area correlated with lower MIC values against *S. aureus* and *E. coli*, consistent with enhanced membrane partitioning.^64,71^ This effect was particularly pronounced in macrocyclic peptoids, where preorganisation of side chains amplifies hydrophobic interactions.

In their study carried out by Frederiksen and colleagues, increasing hydrophobicity through substitution of Ntrp 36 with more hydrophobic monomers (*e.g.*, Ndpe and Nain) was observed to increased RP-HPLC retention times and enhanced antibacterial activity against multiple strains, consistent with strengthened hydrophobic interactions between peptoids and bacterial membranes. However, this relationship was not universal: substitution with Nspe increased hydrophobicity but reduced activity against *S. aureus*, and replacement of Ntrp with Npe conferred no measurable improvement in antimicrobial potency.^20,72^

### 5.2 Halogenation

Halogenation of aromatic side chains provides an effective strategy for modulating peptoid hydrophobicity, polarizability, and self-assembly without altering the underlying backbone architecture.^73^ Introduction of bromine or chlorine substituents onto phenyl or naphthyl moieties increases lipophilicity and can strengthen interactions with the hydrophobic core of lipid bilayers, thereby enhancing membrane activity (72).^74,75^

A recent study examined the impact of halogen substitution on biological activity, revealing that short brominated analogues displayed up to a 32-fold improvement in activity against *S. aureus* and a 16- to 64-fold increase against *Escherichia coli* and *Pseudomonas aeruginosa*, accompanied by reduced cytotoxicity.^53^

**Fig. 15:**
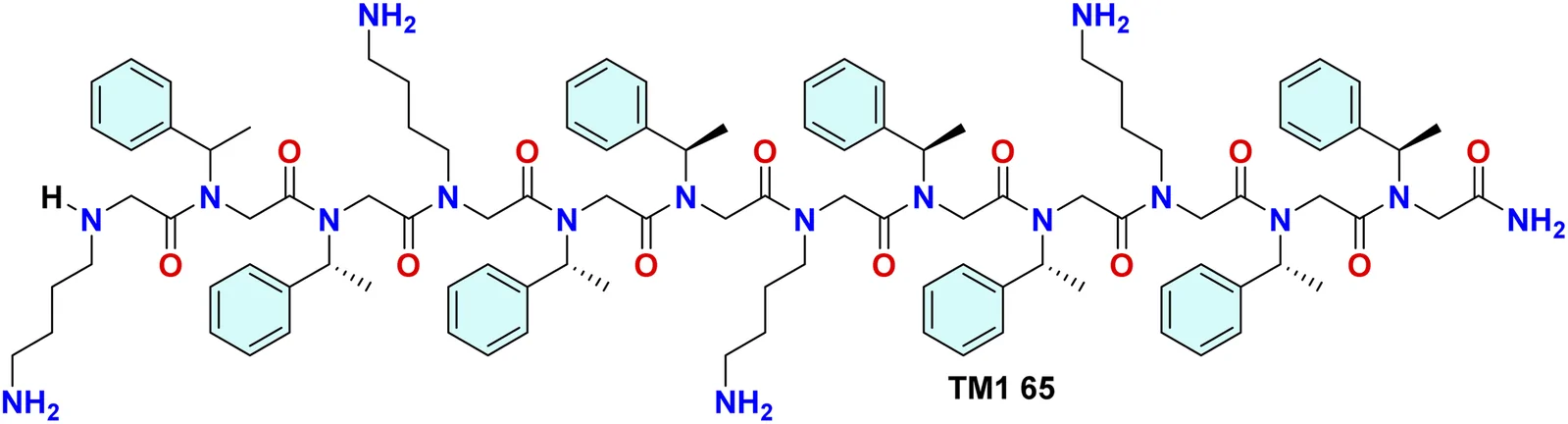
Peptoids TM1.

**Fig. 16:**
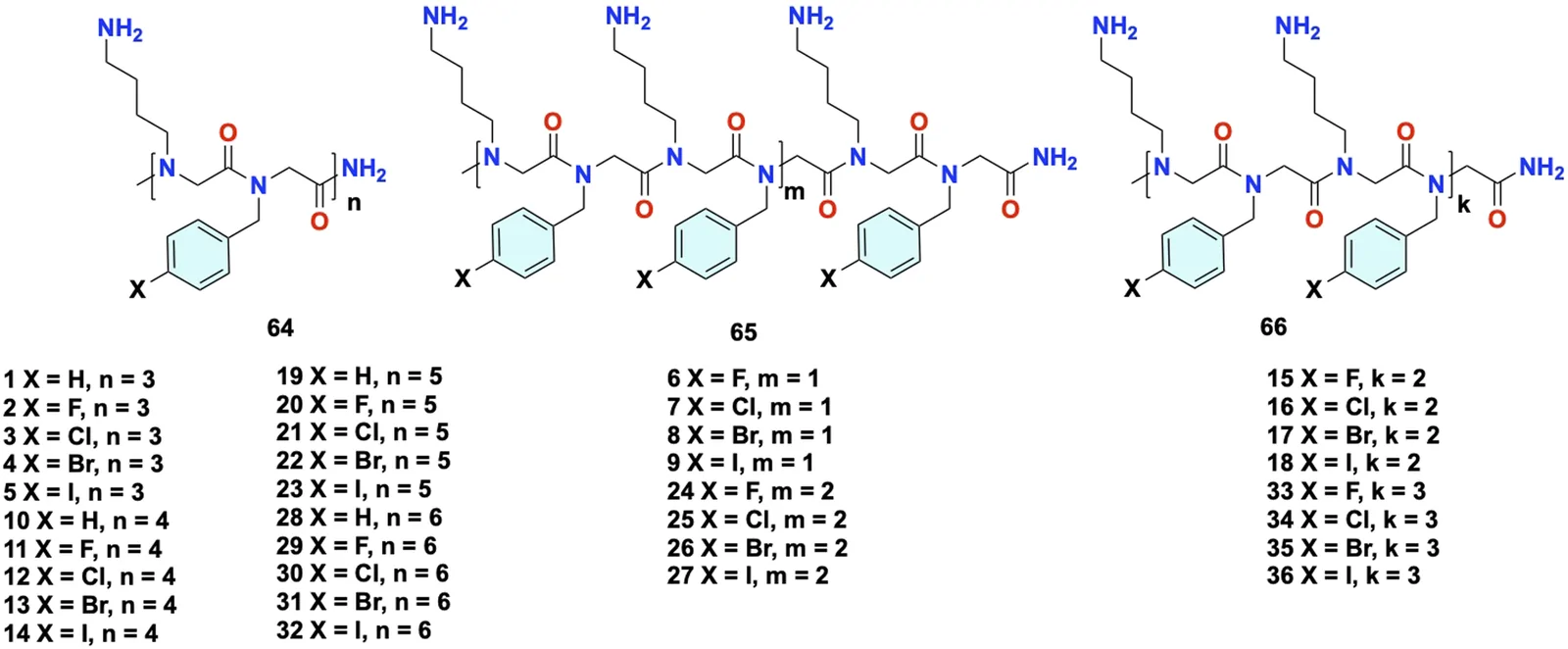
Modulating of peptoid with halogen.

Nielsen *et al.* evaluated peptoids derived from TM1 65 and TM5 57 (Fig. 13 and 15) in which specific aromatic residues were replaced with *para*-brominated analogues.^53^ Halogenated variants (TM2, TM4) generally showed enhanced activity against ESKAPE pathogens, including *Enterococcus faecium* and *Pseudomonas aeruginosa*, sometimes achieving MICs comparable to or lower than TM1 65 while maintaining acceptable haemolysis.^55^ Small-angle X-ray scattering revealed that halogenation promoted formation of larger, more elongated aggregates, consistent with stronger intermolecular hydrophobic interactions, which correlated with shifts in antibacterial potency (Fig. 16).

Antibacterial activity was benchmarked against MSI-78 (pexiganan). To interrogate structure–activity relationships, electron-donating and electron-withdrawing substituents were installed on the phenyl ring and compared in peptoid scaffolds bearing either ammonium or guanidinium terminal groups. Chloro-substituted analogues displayed the highest potency across both Gram-positive and Gram-negative strains. Notably, conversion of terminal amino groups to guanidinium significantly enhanced activity against *S. aureus* 38, irrespective of the aryl substituent (Cl, 2-Cl, F, NO_2_, CH_3_, *tert*-Bu).^76–78^

Enhanced efficacy of halogenated peptoids is consistent with reports that halogen lone-pair electrons increase electrostatic potential, membrane permeability, and phospholipid interactions.^22,73^

## 6 Secondary structure and conformational control

The conformational preferences of peptoids play a crucial role in determining how hydrophobic and cationic residues are displayed to bacterial membranes and thus strongly influence activity. Although peptoids lack backbone N–H groups and therefore cannot form classical peptide hydrogen-bond networks,^65^ their amide bonds can adopt *cis* or *trans* geometries, and side-chain sterics and stereochemistry can bias backbone *φ*/*ψ* angles towards specific conformations. Pre-organisation of an amphipathic surface reduces the entropic penalty for membrane binding and can lead to higher antibacterial potency and improved selectivity, analogous to the behaviour of helical AMPs and therefore potentially also applicable to AMPs mimics.^79^

### 6.1 Helical peptoids as AMP mimics

A landmark contribution to peptoid structural chemistry was the demonstration that incorporation of bulky, α-chiral side chains (*e.g. N*-(*S*-α-methylbenzyl)glycine) biases the backbone towards a right-handed polyproline type I-like helix.^80,81^ Study has also showed that oligomers comprising such residues adopt stable helical conformations in solution, with side chains arranged in a way that can mimic the amphipathic faces of α-helical peptides.^82^

**Fig. 17:**
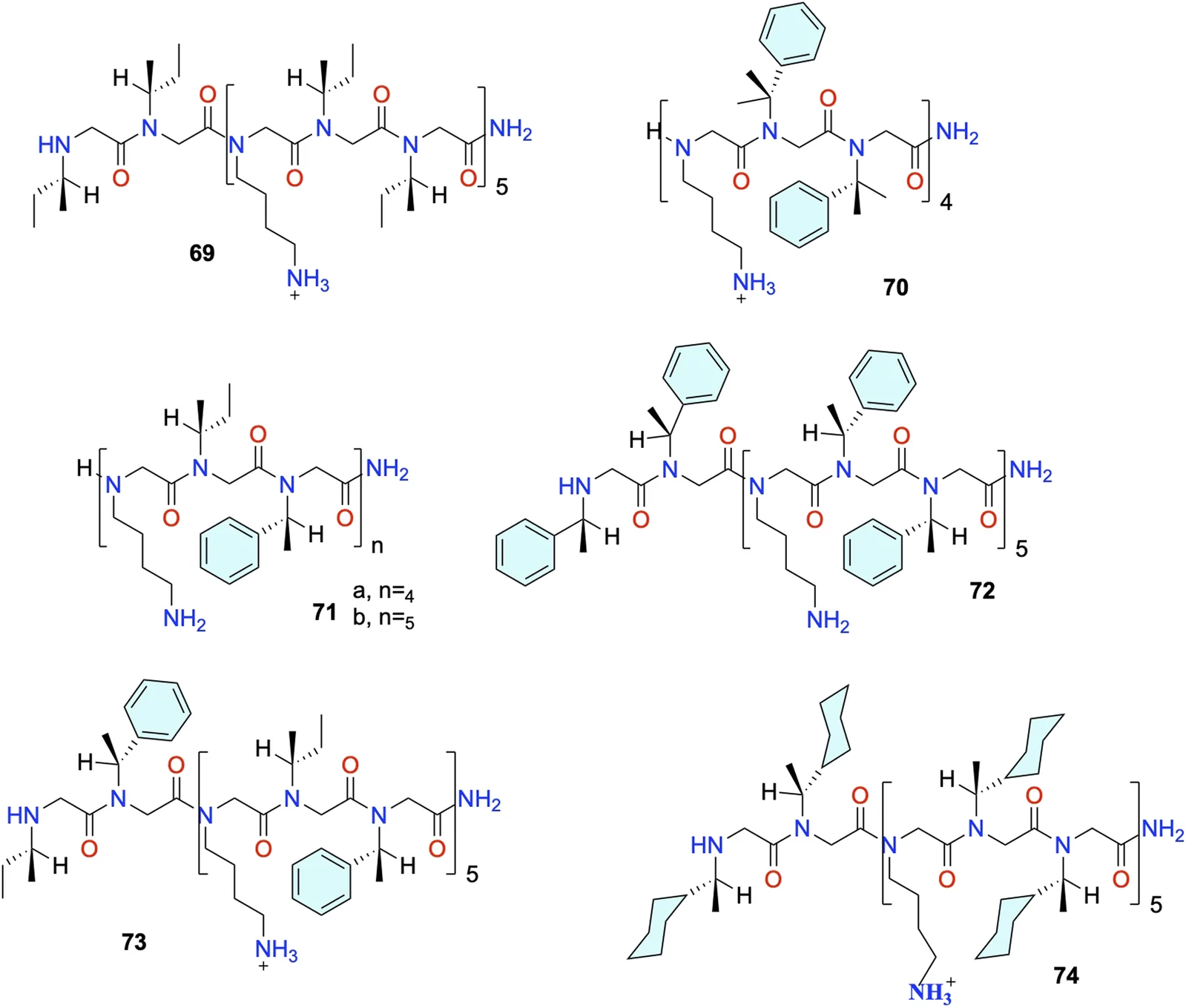
Peptoid mimics of magainin-2 amide.

Helical, cationic peptoid mimics of magainin-2 were rationally designed and synthesised to delineate structure–activity relationships within antimicrobial foldamers. Analogues 69–74 (Fig. 17) incorporated α-chiral hydrophobic residues, predominantly Nspe, in combination with periodically spaced cationic NLys units to enforce facial amphiphilicity. Circular dichroism analyses confirmed that derivatives of peptoid 71 adopted stable helical conformations, particularly in membrane-mimetic vesicles, whereas analogues 69 and 74 exhibited comparatively weak secondary structure, underscoring the importance of side-chain patterning in helix stabilization.^45^

Biological evaluation revealed a pronounced length dependence, with peptoids comprising 12–17 residues displaying the greatest antibacterial activity. Peptoid 70 (12-mer) exhibited the most potent and broad-spectrum profile, achieving low-micromolar MICs against *Escherichia coli* and *Bacillus subtilis*. Similarly, peptoids 71b (15-mer) and 73 (17-mer) demonstrated robust antibacterial efficacy, while shorter congeners were largely inactive, indicating a minimum chain-length requirement for activity. Consistent with membrane-targeting mechanisms, the active analogues generally showed enhanced potency against Gram-positive organisms.

Selectivity was primarily dictated by hydrophobicity. The relatively hydrophilic peptoids 69–71b induced negligible haemolysis, whereas the more lipophilic analogues 72 and 74 (Fig. 17) were strongly haemolytic. Notably, peptoids 71b and 70 achieved an optimal balance between antimicrobial potency and erythrocyte compatibility at biologically relevant concentrations.^45^ Subsequent work extended this approach to pexiganan mimics and other helical scaffolds, further confirming that conformationally pre-organised peptoids can match or exceed the antibacterial performance of their peptide templates while benefiting from superior proteolytic stability.^39^

These studies revealed that the number and placement of helix-inducing monomers must be optimised: too few result in partially disordered backbones and weakened amphipathic presentation, while too many or poorly placed helix promoters can render the molecule overly rigid and less able to adapt to different membrane environments. An intermediate degree of conformational constraint appears to be ideal.

## 7 Advanced peptoid architectures and systems

### 7.1 Peptoid–peptide hybrid and chimeric oligomers

Hybrid peptoid–peptide systems represent a promising approach to combine the biological recognition features of peptides with the stability and tunability of peptoids. In these chimeric oligomers, peptide segments contribute secondary structure and bioactivity, while peptoid residues impart protease resistance and modulate amphipathicity.^80,83^ Early studies demonstrated that incorporating peptoid residues into AMP backbones preserved antibacterial activity while significantly enhancing serum stability.^84^

Recent investigations have shown that hybrid sequences lacking strong helix-inducing constraints often display superior selectivity. For example, sequences with reduced conformational rigidity were found to disrupt bacterial membranes effectively while minimizing damage to erythrocytes, suggesting that excessive structural preorganization may be detrimental to selectivity.^85,86^ These findings highlight the importance of balancing structural order and flexibility in hybrid systems.

Another study also reported a new methodology for mRNA-programmed synthesis of peptoids and peptoid–peptide hybrids by means of translation machinery under the reprogrammed genetic code. Where they initially screened *N*-substituted glycines (rGly) for their single incorporation into a nascent peptide chain and found translation machinery accepts a variety of rGly for elongation^87^

In another study, the authors investigated the effect of systematic peptoid incorporation into the membrane-disruptive undecapeptide KKLLKLLKLLL (K = lysine; L = leucine), with the objective of assessing whether backbone modification could induce a mechanistic transition from membrane lysis toward intracellular targeting. A series of peptide–peptoid hybrids containing increasing numbers of peptoid residues was designed, synthesized, and evaluated for secondary structure, membrane activity, antimicrobial potency, and cytotoxicity.^84^

**Fig. 18:**
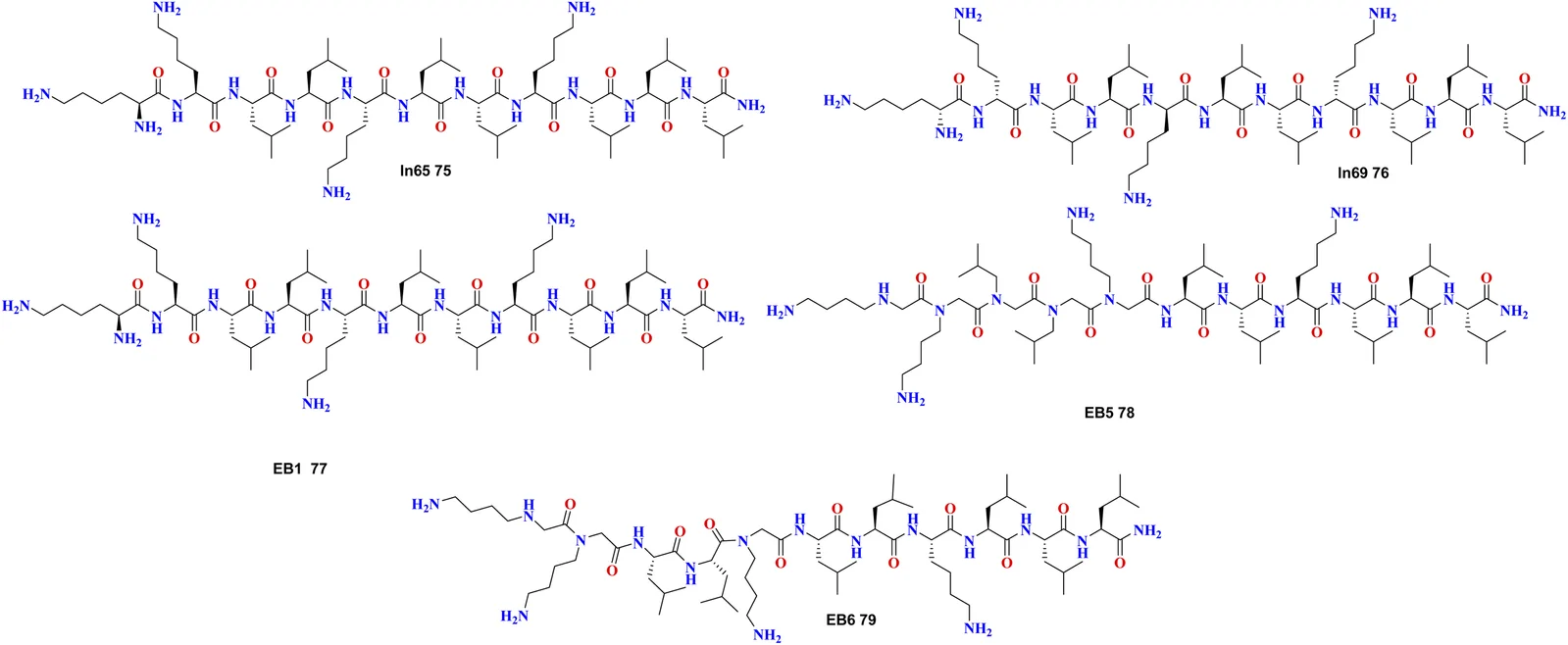
Antimicrobial peptide–peptoid hybrids by Bonvin group.

In that study, Bonvin and co-workers evaluated a series of peptide–peptoid hybrids in full Mueller–Hinton (MH) medium. Three hybrids, EB1 77, EB5 78, and EB6 79, exhibited antibacterial activity against five tested species comparable to the parent AMPs ln65 75 and ln69 76 (MIC = 2–8 µg mL^−1^), while maintaining similar haemolytic profiles (MHC = 250–1000 µg mL^−1^). Time–kill assays showed that EB6 rapidly achieved complete killing of both *E. coli* and *P. aeruginosa* within a few hours, mirroring the parent peptides, whereas EB5 78 produced regrowth of *P. aeruginosa* PAO1 after 3 h, indicating incomplete bactericidal activity (Fig. 18). Structural analysis indicated that hybrids containing up to five peptoid substitutions retained the α-helical conformation of the parent AMPs and preserved membrane-disruptive function.^84^ These analogues demonstrated potent activity against Gram-negative pathogens, including multidrug-resistant *P. aeruginosa* and *Klebsiella pneumoniae*. Notably, partial peptoid incorporation reduced hemolysis and mammalian cytotoxicity, suggesting improved therapeutic selectivity (Fig. 18).

**Fig. 19:**
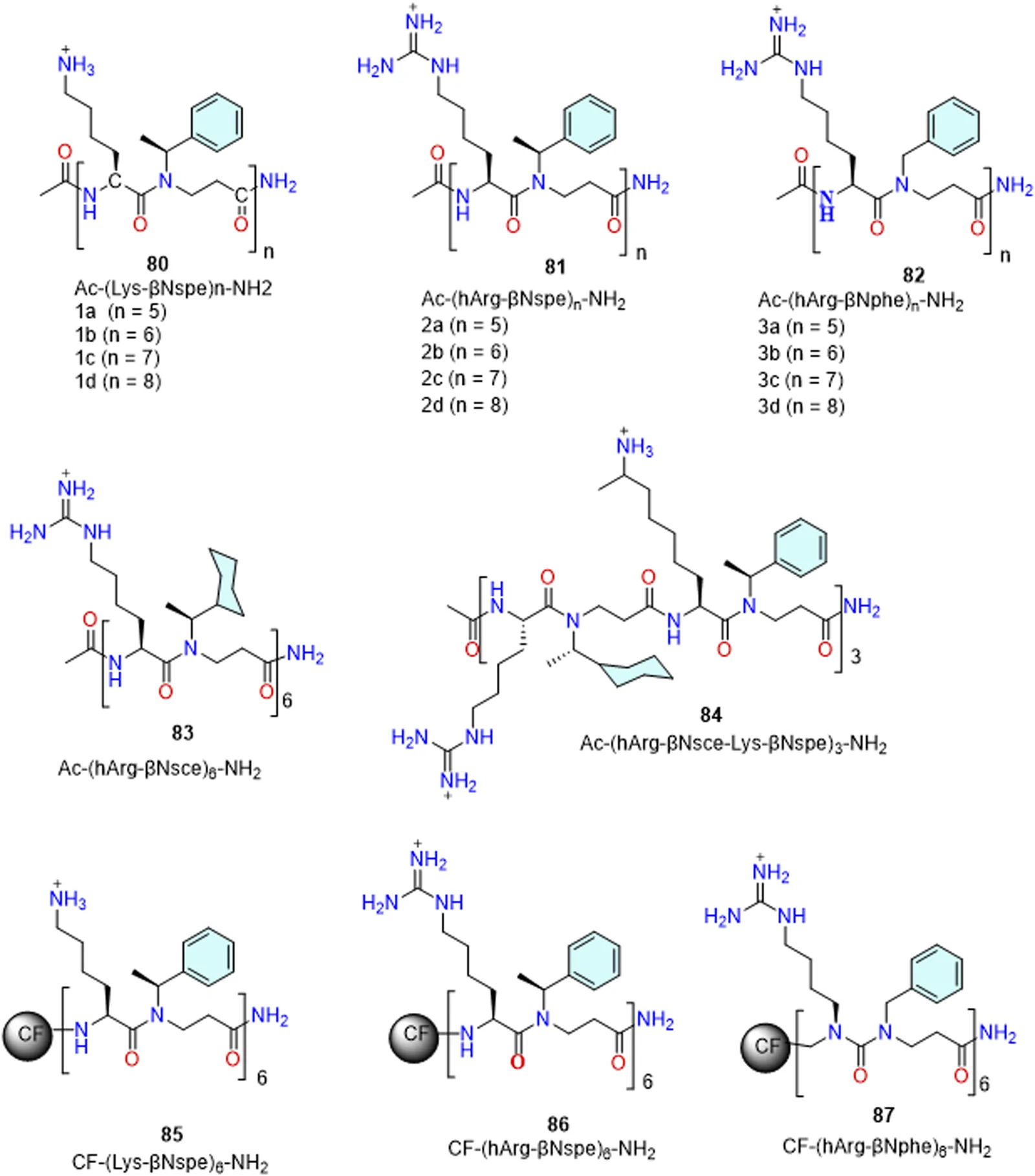
The chemical structures of a series of linear hybrid oligomers comprising alternating cationic and hydrophobic β-peptoid residues.

Peptoid–peptide hybrids represent another avenue: Olsen *et al.* synthesized α-peptide/β-peptoid chimeras that combined the conformational preferences of peptides with the stability of peptoids and observed potent antibacterial activity with good protease resistance.^83^ The authors reported that increasing oligomer length generally enhanced antibacterial potency. Guanidino-substituted analogues in series 81a–d and 82a–d were usually more active than the amino analogues, except against *Pseudomonas aeruginosa*, where compound 80d was more potent than 81d and 82d and showed activity comparable to pexiganan with a markedly higher HC10. Circular dichroism studies indicated greater folding in the α-chiral β-peptoid series 81a–d, and methicillin-resistant *S. aureus* was more sensitive to these oligomers, whereas vancomycin-resistant enterococci, *Escherichia coli*, and *P. aeruginosa* showed no clear dependence on folding. *Candida albicans* was more susceptible to the achiral series 82a–d, which identified 82b as a potential antifungal lead. They also found that *N*-terminal fluorescein labelling in compounds 85–87 reduced activity against all tested organisms. The hybrid oligomers were resistant to proteolytic degradation by chymotrypsin, pronase, and trypsin for up to 50 hours, whereas pexiganan was rapidly degraded. Cytotoxicity studies showed that 80d and 82a were weakly toxic with no significant erythrocyte alterations up to about 100 µg mL^−1^, while 81d and 82d caused membrane alterations at approximately 20 µg mL^−1^. Overall, the hybrids displayed toxicity comparable to or lower than pexiganan and substantially lower than melittin (Fig. 19).^83^

### 7.2. Dimeric, multimeric, and self-assembling peptoids

Beyond monomeric oligomers, multimeric and self-assembling peptoid architectures have attracted growing interest. Dimerization and multimerization increase local charge density and hydrophobic surface area, often leading to enhanced membrane disruption. Several studies have demonstrated that dimeric peptoids exhibit significantly lower MIC values than their monomeric counterparts,^8^ particularly against Gram-negative bacteria with complex outer membranes.^88,89^

Self-assembling peptoids introduce an additional layer of functional complexity amphiphilic peptoids can spontaneously self-assemble into nanosheets, fibers, or micellar structures, which interact cooperatively with bacterial membranes. Whether such assembly helps or hinders is not fixed: small-angle X-ray scattering studies show that aggregation number, and the relationship between the critical aggregation concentration and the MIC, determine whether self-association concentrates the peptoid at the bacterial surface or sequesters it away from the membrane.^53,54,60,90^ Such supramolecular assemblies can enhance local concentration at bacterial surfaces and promote membrane destabilization through collective effects. Importantly, self-assembly can be engineered to be responsive to environmental cues such as pH or ionic strength, offering opportunities for selective activation in infection sites.

### 7.3 Polymer and nanostructured peptoids

**Fig. 20:**
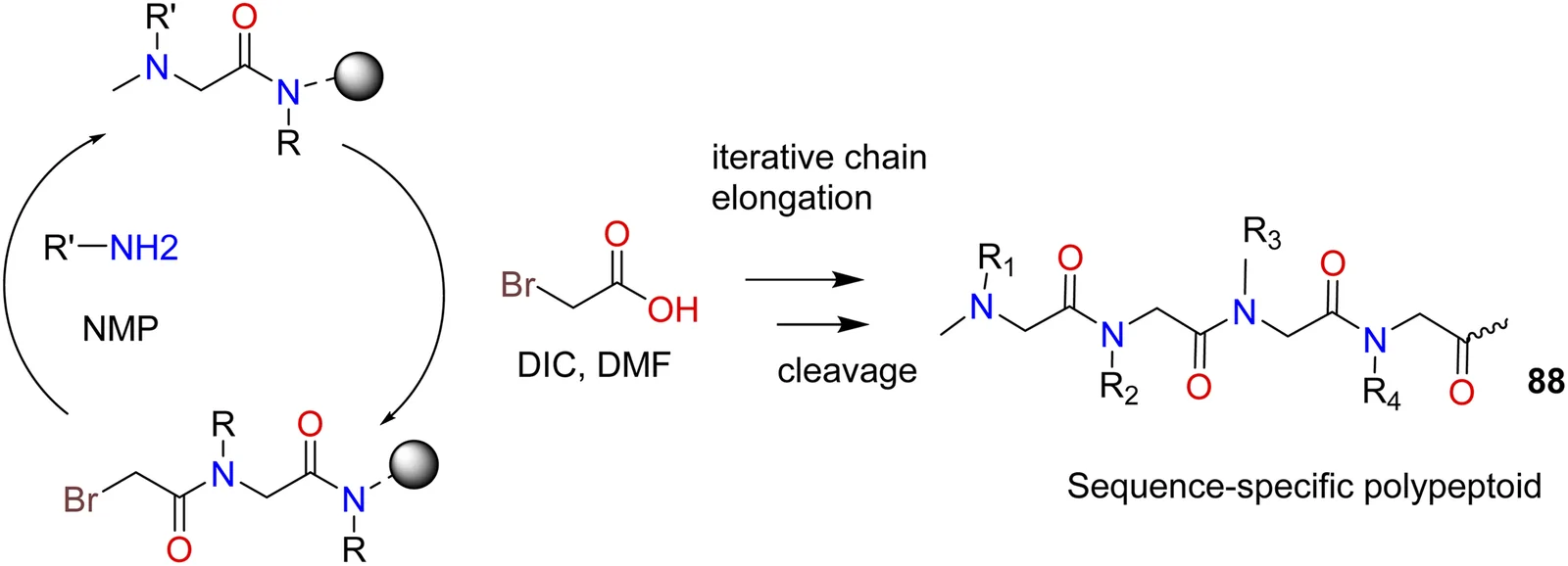
Solid-phase submonomer approached for rapid synthesis of sequence-species peptoids.

Polymeric peptoids and peptoid-based nanostructures represent the most recent frontier in antibacterial peptoid research. α-Peptoid polymers, designed as synthetic mimics of antimicrobial polymers, exhibit broad-spectrum activity and excellent stability. Increasing polymer chain length generally enhances antibacterial efficacy by amplifying multivalent interactions with bacterial membranes; however, excessive length often leads to aggregation and increased cytotoxicity. Recent work has therefore focused on identifying optimal degrees of polymerization that balance potency and selectivity,^91^ and the solid-phase submonomer route remains the enabling method for the rapid, sequence-specific synthesis of these materials (Fig. 20).^24^

Nanostructured peptoids, including peptoid-functionalized nanoparticles and hydrogels, have shown particular promise for biomedical applications.^91^ These systems enable sustained release, localized activity, and improved performance against biofilms. For instance, peptoid-based hydrogels have demonstrated potent antibacterial activity while supporting mammalian cell viability, making them attractive candidates for wound healing and implant coatings.^40,44^

In another example, Ma and colleagues. Synthesized a series of cationic amphiphilic disubstituted polypeptoids *via* ring-opening polymerization (ROP) followed by thiol–ene click chemistry. This class of peptidomimetic polymers featured chiral centers along the backbone and ammonium alkyl *N*-substituents, which promoted the formation of remarkably stable helical structures. Notably, these helices remained stable across a wide range of pH values, temperatures, salt concentrations, and in the presence of denaturing agents.^92^

Comparative antibacterial assays demonstrated that the helical polypeptoids exhibited significantly higher activity against both Gram-negative and Gram-positive bacterial strains than their racemic, non-helical counterparts. Structure–activity relationship analysis revealed that the maintenance of helical conformation, in conjunction with a carefully balanced distribution of cationic charges and hydrophobic residues, was critical for achieving high bacterial selectivity over mammalian cells.

**Fig. 21:**
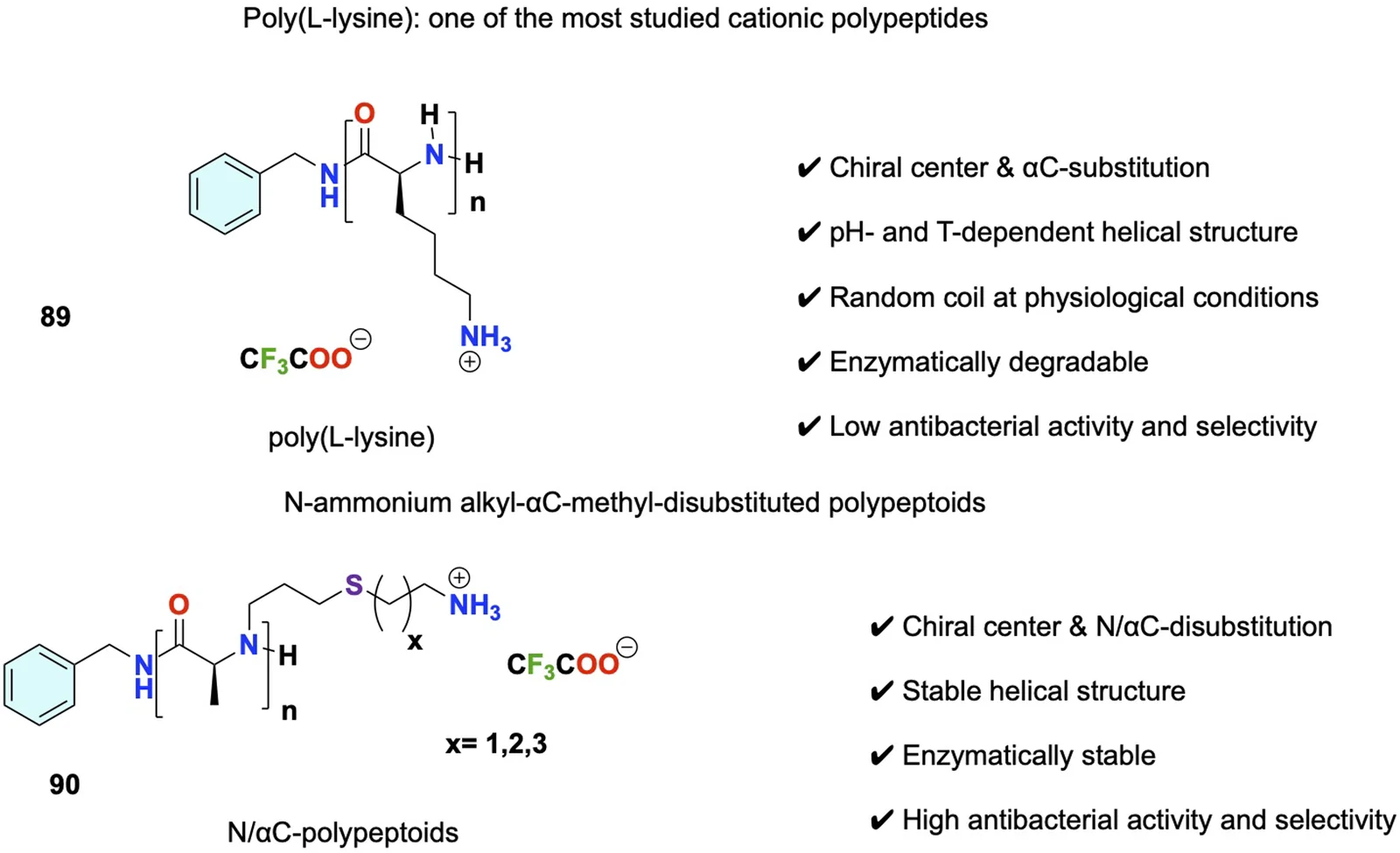
Comparison of L-PLys (a) and *N*-ammonium alkyl-αC-methyl-disubstituted polypeptoids (b).

Furthermore, unlike poly(l-lysine) 89, these disubstituted polypeptoids 90 retained potent antibacterial activity under physiologically relevant conditions, including the presence of salts, human serum albumin (HSA), and protease trypsin, owing to their stable helical architecture and enzymatic resistance.^92^ Collectively, these advances showcase the versatility of peptoids as antibacterial agents. Through precise control over molecular architecture, side-chain chemistry, and supramolecular organization, modern peptoid design has progressed from simple AMP mimics to sophisticated antibacterial platforms capable of addressing contemporary challenges such as antimicrobial resistance and biofilm-associated infections (Fig. 21).

## 8 Challenges and future perspectives

The preceding sections have addressed the structural determinants of antibacterial activity. Table 2 summarises the peptoids presently in use or in advanced development across the medical, medical-device and industrial sectors, together with the architecture, side-chain chemistry, lipid monomer type, reported hydrophobicity index and design features underlying their performance.

**Table 2:** Peptoids in current use or advanced development and their features^a^

Peptoid/peptoid material	Sector & development status	Architecture, length & cationic charge	Side-chain classes present	Lipid/terminal (lipopeptoid) monomer	Reported hydrophobicity index	Structural design features rendering it effective	Reference
(A) Medicine: therapeutic candidates in advanced translational development							
Peptoid 1 (8)	Preclinical broad-spectrum antimicrobial lead	Linear 12-mer; helical, with the NLys-Nspe-Nspe trimer motif repeated four times; net charge +4	Cationic aliphatic (4 × NLys, 4-carbon primary amine); aromatic α-chiral (8 × Nspe). No halogen	None (unlipidated)	RP-HPLC retention time 14.9 min (C18, 0 to 100% MeCN over 30 min, 0.1% TFA); log *D* = +1.21	Threefold cationic/hydrophobic periodicity produces a facially amphipathic helix; the α-chiral, sterically bulky Nspe monomer stabilises that helix through local steric effects. Prevents formation of and detaches MSSA and MRSA biofilms at 1.6 µM, and cleared infection in treated mice within 8 days	93 and 94–96
TM5 (57)	Preclinical antibacterial; identified as a lead against multidrug-resistant ocular pathogens	Linear lipopeptoid: 4-residue core plus an N-terminal alkyl unit; two cationic NLys residues	Cationic NLys; aromatic Nspe; aliphatic tridecyl tail. No halogen	*N*-tridecyl (C13) at the amino terminus	Not reported as a numerical index; compounds ranked by RP-HPLC retention	Shows that N-terminal alkylation restores potency. TM5 had a high therapeutic index, being non-toxic to erythrocytes and to corneal epithelial cells even at 15 to 22 times its MIC. MICs of 7.8 to 31 µg mL^−1^ against ocular *P. aeruginosa* isolates are reported for TM5 and TM18 together, not for TM5 alone	59, 94 and 97
TM8	Preclinical antibacterial and antifungal lead against WHO priority pathogens, including metallo-β-lactamase producers and *Candida auris*	Linear lipopeptoid: 6-residue core plus an N-terminal alkyl unit; two cationic NLys residues	Cationic NLys; aromatic Nspe; aliphatic decyl tail. Non-brominated parent of TM9 and TM10	*N*-decyl (C10) at the amino terminus	Not reported as a numerical index	Alkylation drives micellar self-assembly, and N-terminally alkylated peptoids were reported to cause less toxicity to erythrocytes. Geometric-mean MIC 7.8 µg mL^−1^ against metallo-β-lactamase producers and 5.5 µg mL^−1^ against C. auris; fourfold synergy with ciprofloxacin against NDM-1 *K. pneumoniae*	59, 94 and 97
TM9	Preclinical antibacterial and antiviral candidate (HSV-1, SARS-CoV-2, murine coronavirus)	Linear lipopeptoid: 6-residue core plus an N-terminal alkyl unit; two cationic NLys residues. Core–shell ellipsoidal micelles	Cationic NLys; aromatic Nspe; halogenated aromatic Nspe(p-Br); aliphatic decyl tail	*N*-decyl (C10) at the amino terminus	Not reported as a numerical index	Combines three independent design levers on one scaffold, against murine hepatitis virus strain 1, EC_50_ 4.47 µg mL^−1^ and CC_50_ 17.52 µg mL^−1^, a reported therapeutic index of 3.92	59, 94, 97 and 98
TM10 (also designated MXB-10)	Preclinical antibacterial	Linear lipopeptoid: 6-residue core plus an N-terminal alkyl unit; two cationic NLys residues. Forms a worm-like micelles	Cationic NLys; aromatic Nspe; halogenated Nspe(p-Br); aliphatic tridecyl tail	*N*-tridecyl (C13) at the amino terminus	Not reported as a numerical index	TM10 differs from TM9 only in tail length (C13 *versus* C10), yet the longer tail shifts self-assembly from ellipsoidal to worm-like micelles. The worm-like morphology was associated with reduced antibacterial, antibiofilm and anti-abscess activity against ESKAPE pathogens relative to peptoids forming ellipsoidal or bundled assemblies	59
TM18: TM8 plus one C-terminal NLys	Preclinical antibacterial lead for *P. aeruginosa* and co-infection; *in vivo* activity in a murine skin infection model	Linear lipopeptoid; three cationic NLys residues, one more than TM8. Core–shell ellipsoidal micelles	Cationic NLys; aromatic Nspe; aliphatic decyl tail. No halogen	*N*-decyl (C10) at the amino terminus	Not reported as a numerical index	Adding a single C-terminal NLys to raise net positive charge was applied systematically across the series (TM11 to TM20 are TM1 to TM10 plus one NLys). TM18 reduced dermonecrosis and bacterial burden *in vivo*, altered transcription of resistance, tolerance and biofilm pathways, and with meropenem nearly cleared co-infections	99
Claromer platform lead, MXB-22,510	Medicine: the sponsor's own materials describe the drug-development work as preclinical, with FDA human trials stated in August 2025 to be planned for 2026	Described by the sponsor as a low-molecular-weight, sequence-specific, self-assembling helical foldamer	Declared to be a cationic, amphipathic mimic of the human cathelicidin LL-37; specific monomers not publicly disclosed	Not disclosed	Not disclosed	Functional biomimicry of α-helical LL-37, with membrane-selective disruption of bacterial, fungal and enveloped-viral membranes. The platform derives from the peptoid series in the rows above	100
pB, peptoid mimic of surfactant protein B	Synthetic lung-surfactant replacement; efficacy shown in a rat lavage-induced acute lung injury model	Linear 20-mer; amphipathic helix	Cationic and hydrophobic residues patterned to emulate *S*P-B(1–25); aliphatic C18 terminus	N-terminal octadecylamine (C18)	Not reported as an index; surface activity measured instead	Reproduces the insertion region and the helical amphipathic patterning of native *S*P-B(1–25), with C18 lipidation as an added anchor. Peptoid-enhanced formulations improved outcomes over synthetic lipids alone, and some matched animal-derived surfactant	101
pC, peptoid mimic of surfactant protein C	Synthetic lung-surfactant replacement	Linear 22-mer; membrane-spanning hydrophobic helix	Eight N-terminal residues analogous to *S*P-C(5–12); 14 aromatic hydrophobic residues forming the transmembrane helix; aliphatic C18 terminus	N-terminal octadecylamine (C18), intended to mimic palmitoylation of residues 5 and 6 of human *S*P-C	Not reported as an index; hydrophobic helix length and side-chain chemistry were varied systematically to tune surface activity	An extended aromatic hydrophobic helix substitutes for the aggregation-prone native *S*P-C helix without misfolding, and the C18 chain replaces the natural palmitoyl anchors. Establishes hydrophobic-helix length as an independently tunable design parameter	101 and 102
(B) Medicine: device coatings and biomaterial surfaces							
TM5 and TM18 covalently immobilised on etafilcon A hydrogel contact lenses	Medical device: antibacterial contact lens coating, *in vitro* development stage	Short linear lipopeptoids tethered by carbodiimide (EDC/NHS) chemistry, oxazoline plasma, or plasma ion immersion implantation	Cationic NLys; aromatic Nspe; aliphatic alkyl tail (C13 in TM5, C10 in TM18)	*N*-tridecyl (C13, TM5); *N*-decyl (C10, TM18)	Surface hydrophobicity measured as contact angle: coatings raised it by no more than 23°, without altering net surface charge	Proteolytic resistance of the peptoid backbone overcomes the loss of activity seen with the peptide Mel4, and covalent tethering preserves activity after moist-heat sterilisation. Carbodiimide-bound peptoids inhibited MDR *P. aeruginosa* adhesion by over 5 log_10_ CFU per lens, outperforming melimine, which needed six times the concentration for a 3 log_10_ reduction; lenses were non-toxic to corneal epithelial cells	97 and 103
PMP1	Medical device: passive antifouling coating on TiO_2_ (titanium implant surfaces)	Linear 20-mer peptoid block with a C-terminal DOPA-lysine pentapeptide surface anchor; uncharged	Oligoether, PEG-like *N*-methoxyethyl side chains; catecholic DOPA anchor residues. No aromatic hydrophobe, no halogen	None	Non-ionic and strongly hydrated; hydrophobicity not quantified as log D or as a retention time	The opposite design logic to the cationic amphipathic series: fouling is prevented by steric and hydration exclusion rather than membrane lysis. The methoxyethyl side chain was chosen for its chemical resemblance to the PEG repeat unit, and DOPA provides robust adsorption to metal oxide. Chain length governs durability: the shortest chains fouled with fibroblasts within days while longer chains stayed cell-free for weeks	104
Polysarcosine (pSar) = poly(*N*-methylglycine) brushes	Medical device: antifouling polymer brush. The simplest possible peptoid side chain	Linear polypeptoid brush, surface-grafted. Can be produced in large quantities by *N*-carboxyanhydride polymerisation rather than by solid-phase synthesis	Aliphatic *N*-methyl only. Non-ionic, non-aromatic, non-halogenated	None	Highly hydrophilic; not quantified as a partition index in the cited work	Minimal side-chain volume combined with high backbone hydration gives excellent resistance to non-specific protein adsorption and cell attachment, with grafting density and chain length dominating performance. Reported to reduce *P. aeruginosa* adhesion for weeks *in vitro*	97 and 105
Peptoid two-dimensional nanomembranes (2DNMs), three antiviral lead variants	Medical material: antiviral coatings, protective barriers and prophylactic applications. Laboratory stage	Two-dimensional nanomembranes self-assembled from amphiphilic peptoids; sequence-programmable	Amphiphilic, with the specific monomers not stated in the abstract. Confirm against the full text before submission	Not stated in the abstract	Not reported as an index; amphiphilic balance is the stated design variable	Multivalent mimicry of host-cell membrane receptors blocks viral attachment, giving a nongenomic and therefore mutation-insensitive mechanism. Three leads suppressed influenza a (H1N1) and Sindbis virus across TCID50, plaque, RT-qPCR and immunofluorescence assays while maintaining more than 90% cell viability, acting at early stages of viral entry and propagation	106
(C) Industry: materials, coatings and drug-delivery excipients							
Polysarcosine-functionalised lipids (pSar-lipids) in lipid nanoparticles	Industry: PEG-lipid alternative for mRNA lipid nanoparticles, under active preclinical development	Linear polypeptoid conjugated to a lipid anchor. Chain length and molar fraction were used to tune particle size, morphology and internal structure	Aliphatic *N*-methyl (sarcosine) repeat; non-ionic	Lipid anchor conjugated to the pSar block, with the polymer chain length and lipid molar fraction as the formulation variables	Hydrophilic stealth block; the hydrophilic/lipophilic balance is set by pSar chain length and lipid molar fraction rather than by a reported index	pSar is a polypeptoid based on the endogenous amino acid sarcosine and had previously shown stealth properties comparable to PEG. Formulated with a suitable ionisable lipid, pSar-LNPs gave high transfection potency, higher protein secretion, lower pro-inflammatory cytokine secretion and reduced complement activation compared with PEG-lipid systems	107
Sequence-defined amphiphilic oligopeptoid side chains in PDMS-, PEO- and PS-based block-copolymer coatings	Industry: marine antifouling and fouling-release coatings, at laboratory bioassay stage	Short sequence-controlled oligopeptoids grafted as pendant side chains onto a block-copolymer scaffold	Hydrophilic methoxy or oligo(ethylene oxide) monomers combined with fluoroalkyl, that is halogenated, monomers. The number and position of the fluorinated units along the oligomer is the primary design variable	Fluoroalkyl, surface-segregating monomers act as the hydrophobic terminus	Amphiphilicity set by the hydrophilic to fluorinated monomer ratio and by sequence; surface composition measured by XPS and contact angle rather than by a partition index	Absence of backbone hydrogen-bond donors is the decisive feature: peptoid-functionalised coatings outperform otherwise-identical peptide coatings, because H-bond donors structure interfacial water and cause attached Ulva linza to bind more strongly. The fluorinated moiety is highly surface active and directs film surface composition, so monomer sequence, not composition alone, controls performance	108–110
Nutshell 236 and the Nutcracker therapeutics peptoid nanoparticle platform (lead structure not disclosed as a full sequence)	Industry: peptoid used as the ionisable component of an mRNA delivery nanoparticle. Preclinical, with *in vivo* mouse data. Named commercial platform	Linear sequence-defined peptoid built as a cationic headgroup block joined to a lipophilic tail block. Lead identified by clustering a library of more than 200 oligomers, then optimised by design-of-experiments	Cationic aminoethyl (Aet) and aminopropyl (Apr) monomers; a 1,3-diol terminal monomer (Apd); lipophilic tail monomers forming the second block	Lipophilic tail block, systematically parameterised as a design variable rather than a single fixed chain	Library clustered on predicted physical properties	Headgroup chemistry governs organ tropism: the more basic primary-amine monomers Aet and Apr gave preferential lung expression, whereas the 1,3-diol Apd monomer gave the highest liver expression and selectivity and was carried forward. Nutshell 236 delivered anti-RSV antibody mRNA at serum levels 2-fold above the benchmark ionisable lipid DLin-MC3-DMA, with minimal tolerability or immune-response concerns	111
Antibody-mimetic peptoid nanosheets	Industry: molecular-recognition elements for chemical and biological sensors. Laboratory stage	Free-floating two-dimensional nanosheets self-assembled from peptoids, displaying a high density of conformationally constrained peptide and peptoid loops	Functional loop sequences displayed on the sheet surface; loop chemistry is the tunable variable	None	Not reported as an index	Multivalent two-dimensional display substitutes for antibody architecture while avoiding the poor stability and high production cost of antibodies. The sheets are chemically and biologically stable and serve as substrates for enzymes, so the scaffold tolerates a wide range of displayed sequences	112

a Status is given as of September 2026; no entry is clinically approved.

### 8.1 Key challenges: toxicity, stability and delivery

One of the challenges for antibacterial peptoids is that although many sequences have low toxicity to normal cells, very hydrophobic or highly amphipathic designs can cause hemolysis and can damage the membranes of mammalian cells at high concentrations. Therefore, it is very important to hit the right balance between the cationic charge and hydrophobic character in order to save the antimicrobial potency while at the same time reduce off-target membrane disruption. Side-chain identity, sequence length, and overall amphiphilicity need to be finely tuned to enhance therapeutic selectivity.

Further limitations to clinical development arise from pharmacokinetic challenges. Although peptoids are generally more resistant to proteolytic degradation because of backbone *N*-substitution, this modification alone does not necessarily ensure improved oral bioavailability, tissue distribution, or circulation half-life.

The scalability of the chemical synthesis route is still another challenge. The solid-phase submonomer method makes it possible to produce small molecule libraries efficiently and with a high degree of structural diversity; however, as the sequence length gets longer, the yields go down, and the production of large quantities of long or complicated architectures is very expensive and time-consuming. These constraints of the production thus have a direct impact on the feasibility of translation.

One of the main issues that remains undealt with is the systemic delivery of the drugs. Nanoparticle encapsulation and formulation strategies are being studied, but the real breakthrough has so far only been achieved with systems that are surface-bound or localized. On the other hand, coating medical devices with peptoid-functionalized coatings is a rather advanced and novel idea that has great potential because that way one can even avoid systemic pharmacokinetic barriers.

### 8.2 Future directions and innovations

Rational, data-driven design strategies will drive the next wave of peptoid development. Structure–activity relationship studies are increasingly clarifying how charge distribution, helicity, and hydrophobic patterning influence both potency and selectivity. Computational modeling and machine learning are expected to accelerate optimization, reducing reliance on empirical approaches. Beyond systemic therapeutics, peptoids are emerging as attractive candidates for antimicrobial coatings on medical devices, where their localized action offers significant advantages. Efforts are also expanding into antifungal and antiviral applications, further highlighting the versatility of this platform. By contrast, to the best of our knowledge, no peptoid has yet been applied to a plant pathogen or crop-protection setting in the peer-reviewed literature (Table 2), and agriculture therefore remains an entirely unexplored sector for this class. Ultimately, the clinical translation of peptoids as anti-infective agents will hinge on advances in molecular design, scalable synthesis, and formulation strategies.

## 9 Conclusions

The characteristic backbone of peptoids with their potential antibacterial activity makes them a promising research field for designing varied novel antibacterial structures using simple methods. This review covers the designed antibacterial peptoids with potent activity against both Gram-negative and Gram-positive bacteria, as reported by various groups, exploring the structure–activity relationship. Although major progress has been made in linear, cyclic, short cationic, and lipidated peptoid scaffolds, important translational gaps remain, particularly with respect to standardized potency-toxicity benchmarking, mechanistic validation beyond MIC data, pharmacokinetics, and delivery-oriented formulation. Future work on designing peptoids that target bacterial cells needs to explore new shapes and utilize novel sub-monomers to gain a comprehensive understanding of the activity of these unique therapeutic molecules.

## Author contributions

GB, TAI: writing – original draft, GB, TAI, DB, and NK: revision and editing, MW, NK: supervision.

## Conflicts of interest

The authors declare that they have no known competing financial interests or personal relationships that could have appeared to influence the work reported in this paper.

## Data Availability

No new data were generated for this review. All data discussed are available in the cited publications.

## References

[cit1] Nyembe P. L., Ntombela T., Makatini M. M. (2023). Review: Structure-Activity Relationship of Antimicrobial Peptoids. Pharmaceutics.

[cit2] Ibisanmi T. A., Aribisala J. O. (2022). Evaluation of antioxidant, phytochemicals and antibacterial potential of Mormordica charantia (Linn) against pathogenic bacteria isolated from ready to eat food sold in Akure Metropolis, Nigeria. Bull. Natl. Res. Cent..

[cit3] Dini I., De Biasi M.-G., Mancusi A. (2022). An Overview of the Potentialities of Antimicrobial Peptides Derived from Natural Sources. Antibiotics.

[cit4] Erdem Büyükkiraz M., Kesmen Z. (2022). Antimicrobial peptides (AMPs): A promising class of antimicrobial compounds. J. Appl. Microbiol..

[cit5] Ibisanmi T. A., Olowosoke C. B., Ayeni T. O., Faleti A. I. (2024). A computational exploration of whole genome sequences of Klebsiella pneumoniae ST16 for beta-lactam resistance and the discovery of NDM-1 resistance gene inhibitor. Inform. Med. Unlocked.

[cit6] Andersson D. I., Hughes D., Kubicek-Sutherland J. Z. (2016). Mechanisms and consequences of bacterial resistance to antimicrobial peptides. Drug Resistance Updates.

[cit7] Aslam B., Khalid M. H., Aljasir S. F. (2026). Functional Peptides: Comparing Synthetic and Sequence-Engineered Antibiofilm Pharmaceutics. Pharmaceutics.

[cit8] Bahatheg G. (2024). *et al.*, Dimeric peptoids as antibacterial agents. Bioorg. Chem..

[cit9] Ibisanmi T. A., Jiang X., Willcox M., Kumar N. (2026). Recent advances in computational antimicrobial peptide discovery through big data, modeling, and artificial intelligence and their interplay in ushering the next golden era of drug development. Front. Bioinform..

[cit10] Oliveira Júnior N. G., Souza C. M., Buccini D. F., Cardoso M. H., Franco O. L. (2025). Antimicrobial peptides: structure, functions and translational applications. Nat. Rev. Microbiol..

[cit11] Kuppusamy R., Willcox M., Black D. S. C., Kumar N. (2019). Short cationic peptidomimetic antimicrobials. Antibiotics.

[cit12] Godballe T., Nilsson L. L., Petersen P. D., Jenssen H. (2011). Antimicrobial β-Peptides and α-Peptoids. Chem. Biol. Drug Des..

[cit13] Miller S. M. (1995). *et al.*, Comparison of the proteolytic susceptibilities of homologous L-amino acid, D-amino acid, and N-substituted glycine peptide and peptoid oligomers. Drug Dev. Res..

[cit14] Greco I. (2018). *et al.*, In vitro ADME properties of two novel antimicrobial peptoid-based compounds as potential agents against canine pyoderma. Molecules.

[cit15] Zuckermann R. N., Kerr J. M., Moosf W. H., Kent S. B. H. (1992). Efficient Method for the Preparation of Peptoids [Oligo(N-substituted glycines)] by Submonomer Solid-Phase Synthesis. J. Am. Chem. Soc..

[cit16] Zuckermann R. N. (2011). Peptoid origins. Biopolymers.

[cit17] Simon R. J. (1992). *et al.*, Peptoids: A modular approach to drug discovery. Proc. Natl. Acad. Sci. U. S. A..

[cit18] Knight A. S., Zhou E. Y., Francis M. B., Zuckermann R. N. (2015). Sequence Programmable Peptoid Polymers for Diverse Materials Applications. Adv. Mater..

[cit19] Lau J. L., Dunn M. K. (2018). Therapeutic peptides: Historical perspectives, current development trends, and future directions. Bioorg. Med. Chem..

[cit20] Mojsoska B., Zuckermann R. N., Jenssen H. (2015). Structure-Activity Relationship Study of Novel Peptoids That Mimic the Structure of Antimicrobial Peptides. Antimicrob. Agents Chemother..

[cit21] Seraydarian M. R., Connolly M. D., Zuckermann R. N., Kirshenbaum K. (2026). Native Chemical Ligation of Peptoid Oligomers. Biochemistry.

[cit22] Soumanou N., Lybye D., Hjelmgaard T., Faure S. (2023). Greener peptoid synthesis in additive-free water-based media. Green Chem..

[cit23] Zuckermann R. N., Kerr J. M., Moos W. H., Kent S. B. H. (1992). Efficient Method for the Preparation of Peptoids [Oligo(N-Substituted Glycines)] by Submonomer Solid-Phase Synthesis. J. Am. Chem. Soc..

[cit24] Sun J., Zuckermann R. N. (2013). Peptoid polymers: A highly designable bioinspired material. ACS Nano.

[cit25] Miller S. M. (1994). *et al.*, Proteolytic studies of homologous peptide and N-substituted glycine peptoid oligomers. Bioorg. Med. Chem. Lett..

[cit26] Clapperton A. M., Babi J., Tran H. (2022). A Field Guide to Optimizing Peptoid Synthesis. ACS Polym. Au.

[cit27] Fowler S. A., Blackwell H. E. (2009). Structure–function relationships in peptoids: Recent advances toward deciphering the structural requirements for biological function. Org. Biomol. Chem..

[cit28] Lombardi L., Genio V. D., Albericio F., Williams D. R. (2025). Advances in Peptidomimetics for Next-Generation Therapeutics: Strategies, Modifications, and Applications. Chem. Rev..

[cit29] Ng S. (1999). *et al.*, Combinatorial discovery process yields antimicrobial peptoids. Bioorg. Med. Chem..

[cit30] Gottschalk S. (2013). *et al.*, The antimicrobial lysine-peptoid hybrid LP5 inhibits DNA replication and induces the SOS response in Staphylococcus aureus. BMC Microbiol..

[cit31] Bolt H. L., Cobb S. L. (2016). A practical method for the synthesis of peptoids containing both lysine-type and arginine-type monomers. Org. Biomol. Chem..

[cit32] Wender P. A. (2000). *et al.*, The design, synthesis, and evaluation of molecules that enable or enhance cellular uptake: Peptoid molecular transporters. Proc. Natl. Acad. Sci. U. S. A..

[cit33] Ghosh C. (2014). *et al.*, Small molecular antibacterial peptoid mimics: The simpler the better. J. Med. Chem..

[cit34] Amirkhanov N. V., Bardasheva A. V., Tikunova N. V., Pyshnyi D. V. (2021). Synthetic Antimicrobial Peptides: III—Effect of Cationic Groups of Lysine, Arginine, and Histidine on Antimicrobial Activity of Peptides with a Linear Type of Amphipathicity. Russ. J. Bioorg. Chem..

[cit35] Chongsiriwatana N. P. (2011). *et al.*, Short alkylated peptoid mimics of antimicrobial lipopeptides. Antimicrob. Agents Chemother..

[cit36] Rinki K. (2011). *et al.*, Efficacy of Antimicrobial Peptoids against Mycobacterium tuberculosis. Antimicrob. Agents Chemother..

[cit37] Hoque J., Konai M. M., Sequeira S. S., Samaddar S., Haldar J. (2016). Antibacterial and Antibiofilm Activity of Cationic Small Molecules with Spatial Positioning of Hydrophobicity: An in Vitro and in Vivo Evaluation. J. Med. Chem..

[cit38] Bertrand B., Munoz-Garay C. (2025). Unlocking the power of membrane biophysics: enhancing the study of antimicrobial peptides activity and selectivity. Biophys. Rev..

[cit39] Chongsiriwatana N. P. (2008). *et al.*, Peptoids that mimic the structure, function, and mechanism of helical antimicrobial peptides. Proc. Natl. Acad. Sci. U. S. A..

[cit40] Statz A. R., Park J. P., Chongsiriwatana N. P., Barron A. E., Messersmith P. B. (2008). Surface-immobilised antimicrobial peptoids. Biofouling.

[cit41] Andreev K. (2018). *et al.*, Hydrophobic interactions modulate antimicrobial peptoid selectivity towards anionic lipid membranes. Biochim. Biophys. Acta, Biomembr..

[cit42] Wimley W. C. (2010). Describing the Mechanism of Antimicrobial Peptide Action with the Interfacial Activity Model. ACS Chem. Biol..

[cit43] Chen Y. (2007). *et al.*, Role of Peptide Hydrophobicity in the Mechanism of Action of α-Helical Antimicrobial Peptides. Antimicrob. Agents Chemother..

[cit44] Gamna F. (2025). *et al.*, Biofilm prevention and quorum sensing interference via surface-bound peptoid. Surf. Interfaces.

[cit45] Patch J. A., Barron A. E. (2003). Helical peptoid mimics of magainin-2 amide. J. Am. Chem. Soc..

[cit46] Chongsiriwatana N. P. (2008). *et al.*, Peptoids that mimic the structure, function, and mechanism of helical antimicrobial peptides. Proc. Natl. Acad. Sci. U. S. A..

[cit47] Bicker K. L., Cobb S. L. (2020). Recent advances in the development of anti-infective peptoids. Chem. Commun..

[cit48] Lee J. (2018). *et al.*, Effect of side chain hydrophobicity and cationic charge on antimicrobial activity and cytotoxicity of helical peptoids. Bioorg. Med. Chem. Lett..

[cit49] Shyam R. (2018). *et al.*, 1,2,3-Triazolium-Based Cationic Amphipathic Peptoid Oligomers Mimicking Antimicrobial Helical Peptides. ChemMedChem.

[cit50] Shyam R. (2021). *et al.*, Solution-Phase Synthesis of Backbone-Constrained Cationic Peptoid Hexamers with Antibacterial and Anti-Biofilm Activities. Eur. J. Org. Chem..

[cit51] Guerinot C. (2024). *et al.*, Quaternized 1,2,3-Triazolyl Content and Modulation Potentiate Antibacterial and Antifungal Activities of Amphipathic Peptoids. ACS Infect. Dis..

[cit52] Gimenez D. (2019). *et al.*, Fluorinated Aromatic Monomers as Building Blocks to Control α-Peptoid Conformation and Structure. J. Am. Chem. Soc..

[cit53] Molchanova N. (2020). *et al.*, Halogenation as a tool to tune antimicrobial activity of peptoids. Sci. Rep..

[cit54] Bolt H. L. (2017). *et al.*, Exploring the links between peptoid antibacterial activity and toxicity. MedChemComm.

[cit55] Nielsen J. E. (2022). *et al.*, Self-Assembly of Antimicrobial Peptoids Impacts Their Biological Effects on ESKAPE Bacterial Pathogens. ACS Infect. Dis..

[cit56] Oh J. (2025). *et al.*, Local enhancement of cationic charge density via polyamine side chain incorporation improves the selectivity of antimicrobial peptoids. Eur. J. Med. Chem..

[cit57] Huang M. L., Shin S. B. Y., Benson M. A., Torres V. J., Kirshenbaum K. (2012). A comparison of linear and cyclic peptoid oligomers as potent antimicrobial agents. ChemMedChem.

[cit58] Matthyssen T. (2022). *et al.*, The Potential of Modified and Multimeric Antimicrobial Peptide Materials as Superbug Killers. Front. Chem..

[cit59] Nielsen J. E. (2022). *et al.*, Self-Assembly of Antimicrobial Peptoids Impacts Their Biological Effects on ESKAPE Bacterial Pathogens. ACS Infect. Dis..

[cit60] Nielsen J. E. (2022). *et al.*, Self-assembly of antimicrobial peptoids impacts their biological effects on ESKAPE bacterial pathogens. ACS Infect. Dis..

[cit61] Wardell S. J. T. (2025). *et al.*, A biofilm-targeting lipo-peptoid to treat Pseudomonas aeruginosa and Staphylococcus aureus co-infections. Biofilm.

[cit62] Findlay B., Szelemej P., Zhanel G. G., Schweizer F. (2012). Guanidylation and tail effects in cationic antimicrobial lipopeptoids. PLoS One.

[cit63] Green R. M., Bicker K. L. (2020). Evaluation of peptoid mimics of short, lipophilic peptide antimicrobials. Int. J. Antimicrob. Agents.

[cit64] Shin M.-K., Hyun Y.-J., Lee J. H., Lim H.-S. (2018). Comparison of Cell Permeability of Cyclic Peptoids and Linear Peptoids. ACS Comb. Sci..

[cit65] D'Amato A. (2022). Structure – Bioactivity Relationship in Cyclic Peptoids: An Overview. Eur. J. Org. Chem..

[cit66] Makwana K. M., Mahalakshmi R. (2015). Implications of aromatic–aromatic interactions: From protein structures to peptide models. Protein Sci..

[cit67] Muli C. S., Xie D., Post C. B., Trader D. J. (2025). NMR-Guided Studies to Establish the Binding Interaction between a Peptoid and Protein. J. Am. Chem. Soc..

[cit68] Butterfoss G. L. (2012). *et al.*, De novo structure prediction and experimental characterization of folded peptoid oligomers. Proc. Natl. Acad. Sci. U. S. A..

[cit69] Necelis M. R., Santiago-Ortiz L. E., Caputo G. A. (2021). Investigation of the Role of Aromatic Residues in the Antimicrobial Peptide BuCATHL4B. Protein Pept. Lett..

[cit70] Lee E. (2015). *et al.*, Functional Roles of Aromatic Residues and Helices of Papiliocin in its Antimicrobial and Anti-inflammatory Activities. Sci. Rep..

[cit71] Huang M. L., Shin S. B. Y., Benson M. A., Torres V. J., Kirshenbaum K. (2012). A Comparison of Linear and Cyclic Peptoid Oligomers as Potent Antimicrobial Agents. ChemMedChem.

[cit72] mojo N., Hansen P. R., Björkling F., Franzyk H. (2019). Peptide/Peptoid Hybrid Oligomers: The Influence of Hydrophobicity and Relative Side-Chain Length on Antibacterial Activity and Cell Selectivity. Molecules.

[cit73] Mardirossian M. (2021). *et al.*, Natural and Synthetic Halogenated Amino Acids—Structural and Bioactive Features in Antimicrobial Peptides and Peptidomimetics. Molecules.

[cit74] Mardirossian M. (2021). *et al.*, Natural and Synthetic Halogenated Amino Acids—Structural and Bioactive Features in Antimicrobial Peptides and Peptidomimetics. Molecules.

[cit75] Lindner S. (2026). *et al.*, Strategies to Improve the Lipophilicity of Hydrophilic Macromolecular Drugs. Adv. Healthcare Mater..

[cit76] Bahatheg G. (2025). *et al.*, Functionalized Phenyl Peptoids with Enhanced Antibacterial Potency. ACS Infect. Dis..

[cit77] Cohen H. (2023). *et al.*, Interaction of Pexiganan (MSI-78)-Derived Analogues Reduces Inflammation and TLR4-Mediated Cytokine Secretion: A Comparative Study. ACS Omega.

[cit78] Fukuda de Castilho P. (2025). *et al.*, Biocompatible Guanidine-Functionalized Compounds with Biofilm and Membrane Disruptive Activity Against MRSA. ACS Infect. Dis..

[cit79] Tuerkova A. (2020). *et al.*, Effect of helical kink in antimicrobial peptides on membrane pore formation. eLife.

[cit80] Eastwood J. R. B., Weisberg E. I., Katz D., Zuckermann R. N., Kirshenbaum K. (2023). Guidelines for designing peptoid structures: Insights from the Peptoid Data Bank. Pept. Sci..

[cit81] Armand P. (1997). *et al.*, Chiral N-substituted glycines can form stable helical conformations. Folding Des..

[cit82] Wu C. W., Sanborn T. J., Huang K., Zuckermann R. N., Barron A. E. (2001). Peptoid Oligomers with α-Chiral, Aromatic Side Chains: Sequence Requirements for the Formation of Stable Peptoid Helices. J. Am. Chem. Soc..

[cit83] Olsen C. A. (2010). Peptoid–Peptide Hybrid Backbone Architectures. ChemBioChem.

[cit84] Bonvin E. (2023). *et al.*, Antimicrobial Peptide–Peptoid Hybrids with and without Membrane Disruption. ACS Infect. Dis..

[cit85] Zhang L. (2021). *et al.*, Design and Immunological Evaluation of a Hybrid Peptide as a Potent TLR2 Agonist by Structure-Based Virtual Screening. Front. Cell Dev. Biol..

[cit86] Nam H. Y. (2020). *et al.*, Helicity Modulation Improves the Selectivity of Antimicrobial Peptoids. ACS Infect. Dis..

[cit87] Kawakami T., Murakami H., Suga H. (2008). Ribosomal Synthesis of Polypeptoids and Peptoid−Peptide Hybrids. J. Am. Chem. Soc..

[cit88] Bahatheg G. (2022). *et al.*, Short Tryptamine-Based Peptoids as Potential Therapeutics for Microbial Keratitis : Structure-Function Correlation Studies. Antibiotics.

[cit89] Mothahalli Raju N. K., Paul B., TN L., Bodduna S., Kandukuri N. K. (2025). Sulfur-Controlled Modulation of Peptoid Atropisomeric Foldamers. J. Org. Chem..

[cit90] Yang W., Seo J., Kim J. H. (2023). Protein-mimetic peptoid nanoarchitectures for pathogen recognition and neutralization. Nanoscale.

[cit91] Shabani S. (2024). *et al.*, Synthetic peptide branched polymers for antibacterial and biomedical applications. Nat. Rev. Bioeng..

[cit92] Ma A. (2025). *et al.*, High Antibacterial Activity and Selectivity of Cationic Disubstituted Polypeptoids with Stable Helices and Enzymatic Resistance. ACS Appl. Mater. Interfaces.

[cit93] Chongsiriwatana N. P. (2008). *et al.*, Peptoids That Mimic the Structure, Function, and Mechanism of Helical Antimicrobial Peptides. Proc. Natl. Acad. Sci. U. S. A..

[cit94] Diamond G. (2021). *et al.*, Potent Antiviral Activity against HSV-1 and SARS-CoV-2 by Antimicrobial Peptoids. Pharmaceuticals.

[cit95] Wohlwend J. (2024). *et al.*, Boltz-1 Democratizing Biomolecular Interaction Modeling. BioRxiv.

[cit96] Benjamin A. B. (2022). *et al.*, Efficacy of Cathelicidin-Mimetic Antimicrobial Peptoids against Staphylococcus aureus. Microbiol. Spectrum.

[cit97] Sara M. (2024). *et al.*, The activity of antimicrobial peptoids against multidrug-resistant ocular pathogens. Cont. Lens Anterior Eye.

[cit98] Moule M. G. (2024). *et al.*, Peptide-Mimetic Treatment of Pseudomonas Aeruginosa in a Mouse Model of Respiratory Infection. Commun. Biol..

[cit99] Wardell S. J. T. (2025). *et al.*, A Biofilm-Targeting Lipo-Peptoid to Treat Pseudomonas Aeruginosa and Staphylococcus Aureus Co-Infections. Biofilm.

[cit100] Maxwell Biosciences, 2026, Claromer Technology, https://maxwellbiosciences.com

[cit101] Czyzewski A. M. (2018). *et al.*, Effective in vivo treatment of acute lung injury with helical, amphipathic peptoid mimics of pulmonary surfactant proteins. Sci. Rep..

[cit102] Brown N. J., Wu C. W., Seurynck-Servoss S. L., Barron A. E. (2008). Effects of Hydrophobic Helix Length and Side Chain Chemistry on Biomimicry in Peptoid Analogues of SP-C. Biochemistry.

[cit103] Sara M. (2025). *et al.*, The effect of immobilisation strategies on the ability of peptoids to reduce the adhesion of P. aeruginosa strains to contact lenses. Exp. Eye Res..

[cit104] Statz A. R., Meagher R. J., Barron A. E., Messersmith P. B. (2005). New Peptidomimetic Polymers for Antifouling Surfaces. J. Am. Chem. Soc..

[cit105] Lau K. H. A. (2012). *et al.*, Surface-Grafted Polysarcosine as a Peptoid Antifouling Polymer Brush. Langmuir.

[cit106] Phillips B. (2026). *et al.*, Peptoid-Based Nanosheets Exhibiting Broad Antiviral Activity against Enveloped RNA Viruses. ACS Nano.

[cit107] Nogueira S. S. (2020). *et al.*, Polysarcosine-Functionalized Lipid Nanoparticles forTherapeutic mRNA Delivery. ACS Appl. Nano Mater..

[cit108] Barry M. E. (2019). *et al.*, The Role of Hydrogen Bonding in Peptoid-Based Marine Antifouling Coatings. Macromolecules.

[cit109] Patterson A. L. (2017). *et al.*, Role of Backbone Chemistry and Monomer Sequence in Amphiphilic Oligopeptide- and Oligopeptoid-Functionalized PDMS- and PEO-Based Block Copolymers for Marine Antifouling and Fouling Release Coatings. Macromolecules.

[cit110] Van Zoelen W. (2014). *et al.*, Sequence of Hydrophobic and Hydrophilic Residues in Amphiphilic Polymer Coatings Affects Surface Structure and Marine Antifouling/Fouling Release Properties. ACS Macro Lett..

[cit111] Webster E. R. (2024). *et al.*, Discovery of a Peptoid-Based Nanoparticle Platform for Therapeutic mRNA Delivery via Diverse Library Clustering and Structural Parametrization. ACS Nano.

[cit112] Olivier G. K. (2013). *et al.*, Antibody-Mimetic Peptoid Nanosheets for Molecular Recognition. ACS Nano.

